# Quantifying the randomness of the stock markets

**DOI:** 10.1038/s41598-019-49320-9

**Published:** 2019-09-04

**Authors:** Alfonso Delgado-Bonal

**Affiliations:** National University of Distance Education, Faculty of Business and Economics, Madrid, Spain

**Keywords:** Applied mathematics, Statistical physics, thermodynamics and nonlinear dynamics

## Abstract

Randomness has been mathematically defined and quantified in time series using algorithms such as Approximate Entropy (ApEn). Even though ApEn is independent of any model and can be used with any time series, as the markets have different statistical values, it cannot be applied directly to make comparisons between series of financial data. In this paper, we develop further the use of Approximate Entropy to quantify the existence of patterns in evolving data series, defining a measure to allow comparisons between time series and epochs using a maximum entropy approach. We apply the methodology to the stock markets as an example of its application, showing that the number of patterns changed for the six analyzed markets depending on the economic situation, in agreement with the Adaptive Markets Hypothesis.

## Introduction

The stock markets reflect the interaction of many agents buying or selling a particular index. Influenced by their beliefs and the economic situation, the agents may feel inclined to buy or sell under markets with clear trends or act more erratically in epochs of great uncertainty. Therefore, by quantifying the level of randomness of the stock markets we can obtain insights on the general behavior of the participants.

In general, randomness is defined as the lack of patterns; we would say that a market is somehow predictable if it always follows the same price patterns and totally random if there is no repetition of the patterns and the participants buy or sell without any way to determine their next move.

In this regard, Approximate Entropy (ApEn) is a statistical measure of the level of randomness of a data series which is based on counting patterns and their repetitions. Low levels of this statistic indicate the existence of many repeated patterns, and high values indicate randomness and unpredictability. Even though ApEn was originally developed after the entropy concept of Information Theory^1^ for physiological research^2^, it has been used in different fields from psychology^3^ to finance^4^.

However, the applicability of ApEn has been limited for several reasons. First, it is said that ApEn is a biased statistic because the algorithm looks for patterns in the dataset and, in that process, each template vector is compared with itself. This self-counting can be of importance if the number of observations is low, and it was avoided by developing Sample Entropy (SampEn)^5^, which due to its construction is an approximation of the real value of the information contained in the dataset.

The second limitation is the fact that for both ApEn and SampEn it is necessary to select two input parameters, the embedding dimension *m* and the noise filter *r*. While the meaning of the embedding dimension is simple, the different choices of the noise filter can lead to different results and there is no clear guide for its selection. Larger values of the embedding dimension and lower values of the noise filter lead to sharper characteristics of the patterns but can result in situations where the statistic is undefined.

Finally, the biggest limitation for its use in finance is that both algorithms are relative measures. As they are based on the entropy concept from Information Theory, the results are dependent on the alphabet, i.e., the possible outcomes of the “experiment”, which in this case would be the possible values of the stock markets. For situations where the alphabet is known and constant, it is possible to define an absolute measure of randomness via *def* functions^6,7^. However, in finance, and in real data series in general, we do not know *a priori* the list of possible future values. As it is not possible to assume that the alphabets of different stock markets are the same, the values of ApEn are relative to their alphabet and the comparison of the results might not be accurate. However, the values of ApEn have been used for comparisons in previous research^4,8–11^.

Here we propose a methodology to obtain a statistical measure of randomness which does not require the selection of the noise filter *r* and can be used to compare time series. Based on the observation that the maximum value of Approximate Entropy (MaxApEn) is dependent on (i) the length of the dataset, (ii) the order of the series, and (iii) the alphabet, we overcome all the limitations by determining the ratio of MaxApEn between the data series and shuffled versions of itself. We use this ratio to prove that the stock markets have epochs of total randomness and other epochs with higher levels of predictability, indicating which stock markets are repeating more patterns in a particular moment. The results imply that the behavior of the agents interacting in the market changes and adapts to the different economic situations, as suggested by the Adaptive Markets Hypothesis.

This paper is organized as follows. First, we provide an introduction to the Approximate Entropy algorithm, clarifying with examples the role of the embedding dimension *m* and the noise filter *r*. Once the algorithm has been detailed, we exemplify its limitations using the Spanish stock market and propose a methodology to obtain an absolute measure of randomness. Finally, we apply the new methodology to six different stock markets to draw conclusions about their different levels of randomness. In the Supplementary Information, we validate our results with a similar analysis using Sample Entropy and study different time frames to investigate how different amounts of data affect the randomness of the system.

## Methods

### Introduction to Approximate Entropy

In this section, we review briefly the Approximate Entropy algorithm, including two examples to clarify the meaning of the embedding dimension and the noise filter. A description of ApEn can be found in specialized publications^12^ or in comprehensive tutorials where all the theoretical and practical aspects are reviewed^13^.

Formally, the algorithm performs the following tasks:

Step 1. Select the ApEn parameters, i.e., the length of the compared patterns (embedding dimension *m*) and the tolerance or effective noise filter (*r*).

Step 2. Given the dataset {*u*(*i*)} of length *N*, define the subsequences $x(i)=[u(i),\ldots ,u(i+m-1)]$ and $x(j)=[u(j),\ldots ,u(j+m-1)]$, with *i* being the vector acting as template and *j* being the rest of the subsequences of the data series. Determine the Chebyshev distance $d[x(i),x(j)]$ as the maximum distance of the scalar components.

Step 3. Calculate the correlation integrals, which measure the regularity or frequency, within a tolerance *r*, of patterns similar to a given pattern of length *m*. Using the sequences $x(i)=\{x(1),x(2),\ldots ,x(N-m+1)\}$ for $i\le N-m+1$, calculate ${C}_{i}^{m}(r)$ = (number of $j\le N-m+1$ for which $d[x(i),x(j)]\le r$)/($N-m+1$).

Step 4. Compute the $\varphi$ functions proposed by Eckmann and Ruelle: ${\varphi }^{m}(r)=(N-m+1)\,{\sum }_{i=1}^{N-m+1}\,\mathrm{ln}\,{C}_{i}^{m}(r)$.

Step 5. Finally, determine ApEn as the likelihood that runs of patterns that are close for *m* observations remain close on the next incremental comparison:

– ApEn = ${\varphi }^{m+1}(r)-{\varphi }^{m}(r)$ = average over *i* of ln [conditional probability that $|u(i+m)-u(j+m)|\le r$, given the fact that the previous values fulfill the condition $|u(i+k)-u(j+k)|\le r$ for $k=0,1,\ldots ,m-1$]

The result of the algorithm is a real number. Low ApEn values indicate the existence of patterns, which means that the series is somehow predictable, and high values of ApEn indicate randomness and unpredictability.

**Figure 1:**
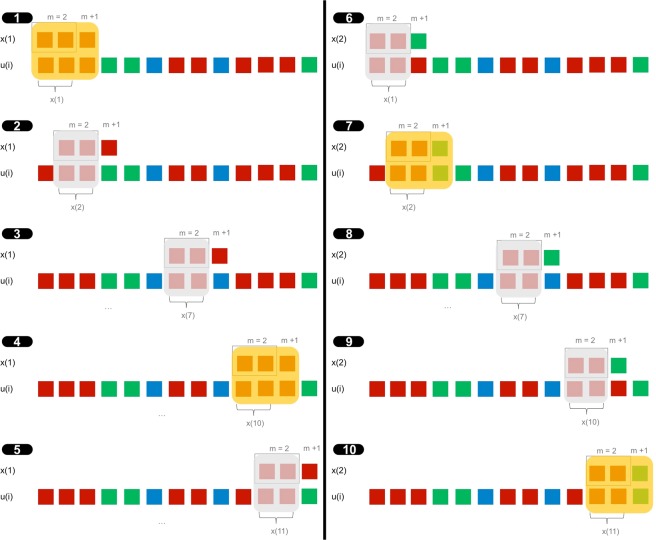
Illustration of the ApEn algorithm for symbolic chains with embedding dimension of *m* = 2.

The idea behind Approximate Entropy is to count how many patterns are repeated throughout a dataset, and two examples will clarify the use of the algorithm. First, we will show how the algorithm is used for symbolic chains, a situation in which the noise filter is not necessary and only the embedding dimension *m* is required. This parameter determines the size of the template window being compared, and *m* = 2 or 3 are usual choices. In the example presented in Fig. 1, the only possible symbols are {*red*, *blue*, *green*}, and we will use an embedding dimension of *m* = 2.

We start with the first illustration on the left side of Fig. 1, taking the first subsequence of size *m*, in this case $x(1)=[red,red]$. Our objective is to check how many times the sequence $[red,red]$ is repeated throughout the series, and how many times after that sequence the next position *m* + 1 is similar to the position after the template vector; in this case the position *m* + 1 = 3 of the template is [*red*].

We have to compare the template with all possible vectors of size *m* = 2 of the dataset in a component-wise way. We start by comparing our template vector $x(1)=[red,red]$ with the first position of the data series *u*. As the first component of *u* is *red*, similar to our template, we compare the next one. As the second one is also similar to the second component of the template vector (*red*), we would say that we have a *possible* vector since all *m* components are similar. We check now the next component, *m* + 1, and since it is similar for the template vector and the dataset (*red*), we would say that it is a *match*. This situation is illustrated in the first image of Fig. 1. This particular case was an obvious match because we were comparing a vector against itself, a situation known as self-counting.

The second image in Fig. 1 continues the process. This time we compare our template vector $x(1)=[red,red]$ with the target vector *x*(2). Comparing component by component, we see how the *m* values are similar, $[red,red]$, having, therefore, a *possible* vector. Thus, we compare the next position to see if it continues the pattern. Unlike the previous case, the component *m* + 1 of the template vector is *red* while the component *m* + 1 of the target vector *x*(2) is *green*, not being a match.

We continue by comparing *x*(1) with *x*(3). The first component is the same, *red*, but the second component of the dataset is of a different color (*green*), not being even a possible vector. Continuing this process, it is easy to see that *x*(7) is the next *possible* vector, as shown in the third image of Fig. 1, but it is not a match. Similarly, the fourth and fifth illustrations show that *x*(10) is a *match* and *x*(11) is a *possible* vector. Therefore, for the first template vector *x*(1) we have accumulated three possible and two match vectors.

We continue the same process but using *x*(2) as a template. The process is illustrated on the right side of Fig. 1, and we count three possible and two match vectors. Once we finish comparing *x*(2), we would continue the process until we use all the vectors as a template once, and then calculate Approximate Entropy as:

1 $$ApEn(m,r,N)\simeq -\,\frac{1}{N-m}\,\mathop{\sum }\limits_{i=1}^{N-m}\,\log \,\frac{{\sum }_{j=1}^{N-m}\,[{\rm{number}}\,{\rm{of}}\,matc{h}_{i}]}{{\sum }_{j=1}^{N-m}\,[{\rm{number}}\,{\rm{of}}\,possibl{e}_{i}]}$$

As we see in Equation 1, ApEn is constructed as a conditional probability between the number of *match* and the number of *possible* vectors. If the patterns are repeated and the position *m* + 1 is often similar to the template vector, we would have a high number of *match*, making the ratio *match*/*possible* close to one and the logarithm close to zero, having ApEn a low value, indicating repeatability and the existence of patterns. On the other hand, if there are no patterns in the dataset and the position *m* + 1 is not usually similar, the negative value of the logarithm would be higher and ApEn would have a high value, indicating randomness. Larger values of *m* imply larger template vectors, being more difficult to find patterns since the conditional probabilities would be more restrictive.

The situation described in Fig. 1 is valid for symbolic chains or for those situations where the alphabet is well defined, like binary chains {0, 1}, a dice {1, 2, 3, 4, 5, 6}, or a decimal number {0, 1, 2, 3, 4, 5, 6, 7, 8, 9} for example. In these situations, it is also possible to determine the maximum randomness of the system, i.e., the maximum value that ApEn could have in those experiments. Pincus and coauthors proved that in those situations the maximum entropy tends to log *k*, with *k* the alphabet of the system, being log 2 for the mentioned binary chain or log 10 for the decimal situation^6^.

**Figure 2:**
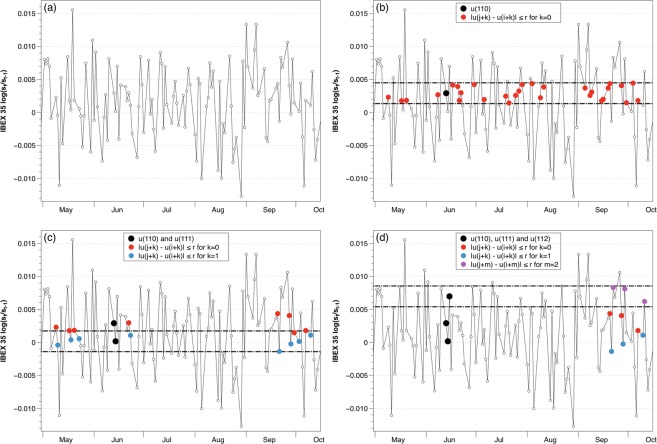
Steps to determine the conditional probabilities of Approximate Entropy for real data considering *m* = 2 and *r* = 0.25*σ*. (**a**) IBEX 35 for 2005 open daily data transformed as log return. (**b**) Red points verify that $d[x(i+k),x(j+k)]\le r$ for $k=0$. (**c**) Blue points verify that $d[x(i+k),x(j+k)]\le r$ for $k=1$. (**d**) Purple points verify that $d[x(i+m),x(j+m)]\le r$ for $m=2$.

Once the role of the embedding dimension has been clarified, we analyze in the next example the role of the noise filter *r*. Figure 2 exemplifies the general situation with real data experiments where the alphabet is unknown a priori, and the example is similar to that found in^13^, where a computer code in R programming language is given to calculate ApEn. For this example, we will use data from the Spanish stock market IBEX 35 for 2005, plotted for a few months after the $\log ({s}_{t}/{s}_{t-1})$ transformation in Fig. 2a. The standard deviation of this series is *σ* = 0.0063, and the noise filter *r* is usually selected as a value in the range $r=[0.1\sigma ,0.25\sigma ]$. For this example, we will use *m* = 2 and *r* = 0.25*σ* = 0.001575.

As in the previous example, we would compare each vector of size *m* with all the other vectors of the sequence. Let us focus on using the vector *x*(110) = [*u*(110), *u*(111)] = [0.00290, 0.00016] as an example of template vector, whose first component is marked as a black dot in Fig. 2b. The parameter *r* determines the dashed lines in that figure, calculated as $u(110)\pm 0.25\sigma$, in this case the range comprehended in [0.004475, 0.001325]. Only the points within that region verify the condition established in the Step 2 for the first component, $|u(j)-u(i)|=|u(j)-u(110)|\le r$, with *u*(*i*) being the first component of the template vector and *u*(*j*) being the first component of all other posible vectors of size *m*. We have marked in red color those points that meet the requirements.

The next step is to check the second component of the template vector, in this case, $u(111)=0.00016$. Once again, the selected value of *r* will determine the range, illustrated with dashed lines in Fig. 2c. In this occasion, we check which points following a red point verify the condition $|u(j+1)-u(i+1)|=|u(j+1)-u(111)|\le r$; we have marked in blue color the points that satisfy the condition. Since those red-blue vectors are similar to the template vector within a tolerance *r*, only those would be our *possible* vectors. In our example, we count nine *possible* vectors, including the template vector.

Finally, we check how many of those possible vectors continue their pattern in a similar way to the template vector. To do so, we check the position *m* + 1 and impose the condition $|u(j+m)-u(i+m)|=|u(j+2)-u(112)|\le r$. We have marked those points following a blue point that meet the requirement in purple color in Fig. 2d. We can see that, out of the nine *possible* vectors, only four are a *match* and share the same pattern of going down and then up.

Once we have checked that template vector, we would select the next vector of size *m* and carry out the same evaluation, continuing the procedure until every vector has served as a template once. Finally, once all the vectors have been used as a template, we would follow the simple calculations in Steps 3 to 6 to calculate the conditional probabilities and determine the value of ApEn.

### MaxApEn

In the previous example, we have seen that the noise filter *r* determines the probability of finding similar patterns, conditioning the number of *possible* and *match* vectors. As *r* is determined as a fraction of the standard deviation of the data, different data sets provide different measures of the noise filter. Mathematically, that fact means that the data may have different alphabets, i.e., the list of possible prices is different and unknown to the researcher beforehand.

**Figure 3:**
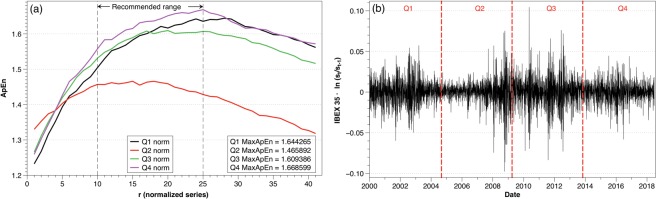
Analysis of IBEX 35 from 2000 to 2018 divided into four quarters Q1 (January 2000–September 2004), Q2 (September 2004–April 2009), Q3 (April 2009–November 2013), Q4 (November 2013–June 2018). (**a**) ApEn for the four quarters of the IBEX 35 data series for different values of *r* as a percentage of the standard deviation of each series. The dotted lines represent the recommended range [0.1*σ*, 0.25*σ*]. (**b**) IBEX 35 daily data presented after the log-ratio transformation from 2000 to 2018 divided into four quarters.

Furthermore, depending on the choice of *r* the values of ApEn and its relative consistency can be different. When *r* increases from 0 to 100*σ*, the value of ApEn increases until it reaches a maximum, decreasing monotonically after it. In many situations, the maximum lies within the recommended range, but it is not always the case. Figure 3a shows the behavior of ApEn as a function of *r* for four series of normalized data, corresponding to the Spanish stock market IBEX 35 from 2000 to 2018 partitioned in four sections, as plotted in Fig. 3b: Q1 (January 2000–September 2004), Q2 (September 2004–April 2009), Q3 (April 2009–November 2013) and Q4 (November 2013–June 2018).

We make different observations from the figure. First, these sequences do not maintain relative consistency, i.e., the lines cross at certain points. It implies that if one uses a value of $r=0.1\sigma$ the classification of the randomness of the series would be Q4 > Q3 > Q1 > Q2, whereas if we select $r=0.25\sigma$ the classification would be Q4 > Q1 > Q3 > Q2. Both values of *r* are in the recommended range [0.1*σ*, 0.25*σ*] but provide different interpretations.

This situation occurs because we are comparing series with different alphabets. For two datasets generated by the same process, it is possible to compare their ApEn values directly because their alphabets are the same. In reality, what we are doing is comparing the ApEn of each dataset with the maximum entropy of that alphabet, but as the maximum entropy is the same because the generating process is the same, the comparison between the two datasets can be done directly. However, it must be noticed that, in general, the comparisons are done between each dataset and its maximum entropy, as Pincus and coauthors did with the *def* functions.

To avoid this problem of which value of *r* should be selected, the use of the maximum value of ApEn (MaxApEn) has been proposed to compare sequences. MaxApEn quantifies the greatest difference in information between the segments of length *m* and *m* + 1, and the selection of *r*_*max*_ allows for greater signal complexity than other values of *r*^14^. Simulations and research with real clinical data have validated the results^15^, using that choice with a “heuristic” justification, allowing a better discrimination capacity and proving its superiority as a regularity statistic^16,17^.

In general, the value of *r* corresponding to the maximum ApEn increases as the number of data decreases, varying with the complexity of the sequence and not always being constrained to the recommended range. Although an automatic method to determine the value of *r*_*max*_ has been proposed^18^, in this paper, we have followed a more traditional approach when executing the calculations by performing iterations for each value of *r* from 0.01 to 0.99 in steps of 0.01 and selecting the maximum value.

The maximization of the entropy provides us with a hierarchy to classify the degree of randomness of each period, eliminating the arbitrariness in the choice of *r*; however, ApEn is a relative measure and the alphabets (the lists of prices) of the four series considered in Fig. 3 may not be the same. Thus, the use of MaxApEn is not sufficient for the comparison of different time series.

### Bootstrapping

In the symbolic example explained in the previous section, the situation of total randomness (the maximum entropy of the system) was determined asymptotically by log *k*, being *k* the base of the alphabet (binary, decimal, etc.). If the maximum entropy is known, then it is possible to define the *def* functions as the difference between the maximum theoretical randomness and the ApEn value^7^, and use that measure to compare systems with the same alphabet; it would be an absolute measure of randomness.

For the stock markets, however, to obtain such an absolute measure it would be necessary to know all the possible future prices. Moreover, when comparing two stock markets or even two sequences of data from the same market at different times, we would have to assume that the alphabet is the same and the generating process of those data remains unchanged, i.e., that we are under the same economic situation and there is a single model which generates data for all the stocks markets.

In Fig. 3b we showed the Spanish stock index, IBEX 35, under the log-ratio transformation from 2000 to 2018. It is clear that, even though it is the same market, the values (and therefore the alphabet) change dramatically, and the comparison of different moments would lack meaning since the maximum values of entropy at each moment would be different. That is the reason why calculations of ApEn are relative and direct comparisons between epochs or indexes are not possible.

Since we do not know the alphabet, a way to calculate the maximum randomness of the series is to shuffle the data a sufficient number of times to obtain representative values of the randomness of the shuffled sequences, a technique known as resampling or bootstrap sampling^19^. This approach of using Monte Carlo simulations to generate sequences with the same number of points but different organizations is widespread when it is not possible to obtain analytical expressions. If the original data series is totally random, the number of repeated patterns would be low and comparable with the number of patterns that may exist in a shuffled version of the data (high ApEn value). If, on the other hand, there is a specific order in the original series, we would find more repeated patterns in that sequence than in its shuffled version (low ApEn).

**Figure 4:**
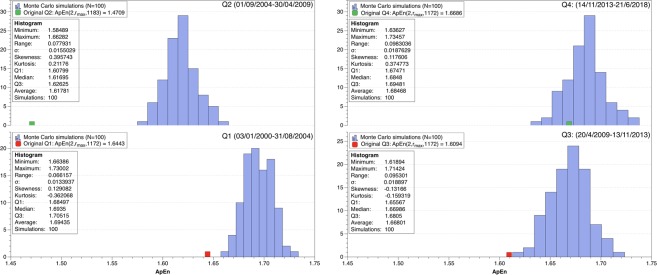
Left: MaxApEn of the periods Q1: 2000–2004 (bottom) and Q2: 2004–2009 (top), and MaxApEn of their shuffled versions for 100 simulations. Right: MaxApEn of the periods Q3: 2009–2013 (bottom) and Q4: 2013–2018 (top), and MaxApEn of their shuffled versions for 100 simulations.

We illustrate this situation in Fig. 4, where we have calculated the value which maximizes ApEn (MaxApEn_*original*_) for each of the four analyzed periods. Then, using the same data series (same alphabet), we have shuffled the values 100 times and determined ApEn as a function of *r*, selecting the value for which it is maximized (histograms of MaxApEn_*shuffled*_). If the alphabets were the same for the four periods, the values of the histograms would be comparable. However, it is clear from Fig. 4 that each analyzed sequence of the IBEX 35 has a different histogram and therefore a different maximum entropy.

At this point, we can compare each original series with its own shuffled versions, given that they have the same alphabet and their reference point is based on the maximum value of ApEn. In Fig. 4 we observe that the first period Q1 is almost compatible with a totally random organization since the value of MaxApEn_*original*_ is very close to its histogram of MaxApEn_*shuffled*_. However, the second quarter, Q2, presents a different situation where the distance from its histogram is considerable. In Q3 the market behaves almost randomly, and finally, in Q4, it is completely random, with the value of MaxApEn of the original series similar to the values of the shuffled series, meaning that the few patterns that exist can be considered accidental.

The problem arises when we try to make a comparison between sequences, for example, Q1 vs. Q3. If we look not only at MaxApEn_*original*_ but also at their shuffled series in Fig. 4, we see that with a value of MaxApEn = 1.64 we would not consider that the Q1 series is random but we would consider that Q3 is. Also, giving another example, a value of MaxApEn = 1.60 in Q2 would be catalogued as random while the same value in Q4 would not be; if the generating process of two series is different (different economic conjuncture with different volatility for example), their alphabets are different and they can not be compared only by looking at their MaxApEn values.

### The Pincus Index

To make the results comparable between series, we realize that the important measure is the distance between MaxApEn_*original*_ and the histogram of MaxApEn_*shuffled*_, and we calculate the ratio MaxApEn(original)/MaxApEn(Monte Carlo), providing a comparable index between series with the same number of points but different alphabets. This proportion of the degree of randomness is zero for completely ordered systems and one (or greater than one) for totally random systems. With this definition, low values of the ratio have the same meaning as low ApEn values, implying that the original series presents predictability and there are patterns, whereas high values imply randomness. We call this measure *Pincus Index (PI)*.

Instead of using the euclidean metric like in the *def* functions when the alphabet is well defined, we have defined the index as a ratio. As we are doing comparisons with different alphabets, the use of the euclidean metric could classify the systems incorrectly: for example, a euclidean difference of *def*(ApEn) = 0.5 has a different meaning in binary chains (where the maximum entropy is log 2 $\simeq$ 0.693) than in decimal chains (where the maximum entropy is log 10 $\simeq$ 2.303). The advantage of using the ratio is that it measures the distance to the maximum entropy situation as a proportion independent of the alphabet.

In Fig. 4 we showed the results of the original sequences and the Monte Carlo analysis of bootstrapped sequences. To determine statistically whether a series is compatible with its shuffled version or not, we use the empirical cumulative distribution function to determine the percentiles of the histograms. We used the median value (50% percentile) of the empirical distribution (histogram) to calculate the value of the Pincus Index, and the 5% and 95% percentiles of the empirical cumulative distribution function to calculate the extremes of the index (error bars in the following figures). It is possible, therefore, that the value of the Pincus Index is greater than one; it simply implies that the MaxApEn values of the tails of the distribution would also be compatible with a random situation.

The Pincus Index provides a way to quantitatively compare different series simply by comparing their values. In that sense, it is an absolute statistical measure of the degree of randomness of the system that characterizes how far a series of data is from a situation statistically compatible with total randomness. We will say that a Pincus Index equal to or greater than one verifies a completely random organization, whereas if the index and its error bars are lower than one, the dataset presents order and patterns; the lower the index, the greater its divergence from randomness.

## Results

In this section, we analyze the randomness of six different stock markets since 1990. The statistical characterizations of the datasets are presented in Table 1, as well as the results of normality and stationarity tests. The results of the Jarque-Bera test indicate that the null hypothesis of normality is rejected for all markets, showing the expected non-normality for daily stock returns, and the Dickey-Fuller and KPSS unit root tests indicate stationarity in mean and variance, induced by the transformation of the logarithm of the returns. The detailed results of these tests, as well as their correlograms and periodograms, are shown in the Supplementary Information for each market.

**Table 1:** Statistical description of the datasets used in this work.

	IBEX 35	FTSE 100	NASDAQ	S&P 500	Hang Seng	Nikkei 225
Number of data	6947	7205	7203	7203	7064	7033
Mean	0.0002	0.0002	0.0004	0.0003	0.0003	0.0000
Median	0.0006	0.0003	0.0012	0.0006	0.0006	0.0000
Minimum	−0.0980	−0.0927	−0.1068	−0.0912	−0.1221	−0.1032
Maximum	0.1045	0.1040	0.1402	0.1014	0.1382	0.1243
Standard Deviation	0.0136	0.0112	0.0147	0.0108	0.0156	0.0139
Skewness	−0.2431	0.0040	−0.3105	−0.2861	−0.1690	0.0112
Kurtosis	4.2556	7.0473	7.6995	8.4851	7.1558	4.6046
5% percentile	−0.0219	−0.0171	−0.0225	−0.0167	−0.0238	−0.0219
95% percentile	0.0204	0.0169	0.0206	0.0159	0.0231	0.0212
Jarque-Bera	5310.5	14909.6	17908.1	21706.2	15105.1	6213.4
Dickey-Fuller	0.0706	0.0000	0.0000	0.0000	0.0000	0.0000
KPSS (p-value)	>0.10	>0.10	>0.10	>0.10	>0.10	>0.10

It is clear from Table 1 that each market is different and their statistical properties are unique. Even after transforming the data to induce stationarity, there is no reason to assume that their alphabets are similar. Thus, instead of comparing their MaxApEn levels directly, we compare each market with its maximum entropy and determine their Pincus Index to draw conclusions about their randomness.

**Figure 5:**
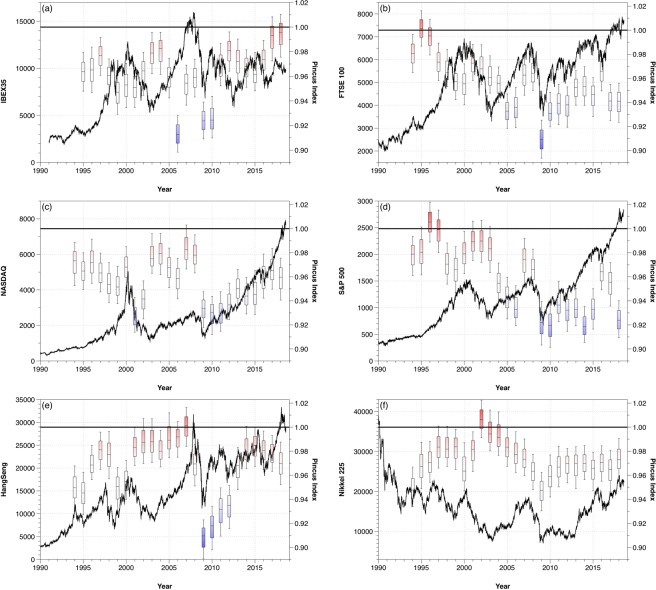
Evolution of the Pincus Index considering information from the previous four years and rolling the window every year.

The amount of information and the randomness of a dataset depends on the number of points considered. In Fig. 5 we show the Pincus Index for six different stock markets, corresponding to Spain (IBEX 35), UK (FTSE 100), USA (NASDAQ and S&P 500), Hong Kong (Hang Seng) and Japan (Nikkei 225). We have considered sequences of four years of opening daily data and rolled the window every year, calculating the Pincus Index of the log-ratio data series. In this way, we can investigate the evolution of the different markets and we observe that, depending on the date, the markets behaved completely random or had repeated patterns.

From the evolution of the PI presented in Fig. 5 we can see that, for example, the FTSE 100 and the S&P 500 behaved like completely random markets in 1996, but both markets verified the existence of patterns in 2018. In 2018, however, other markets which showed patterns in 1996, such as the IBEX 35, had a random behavior.

**Figure 6:**
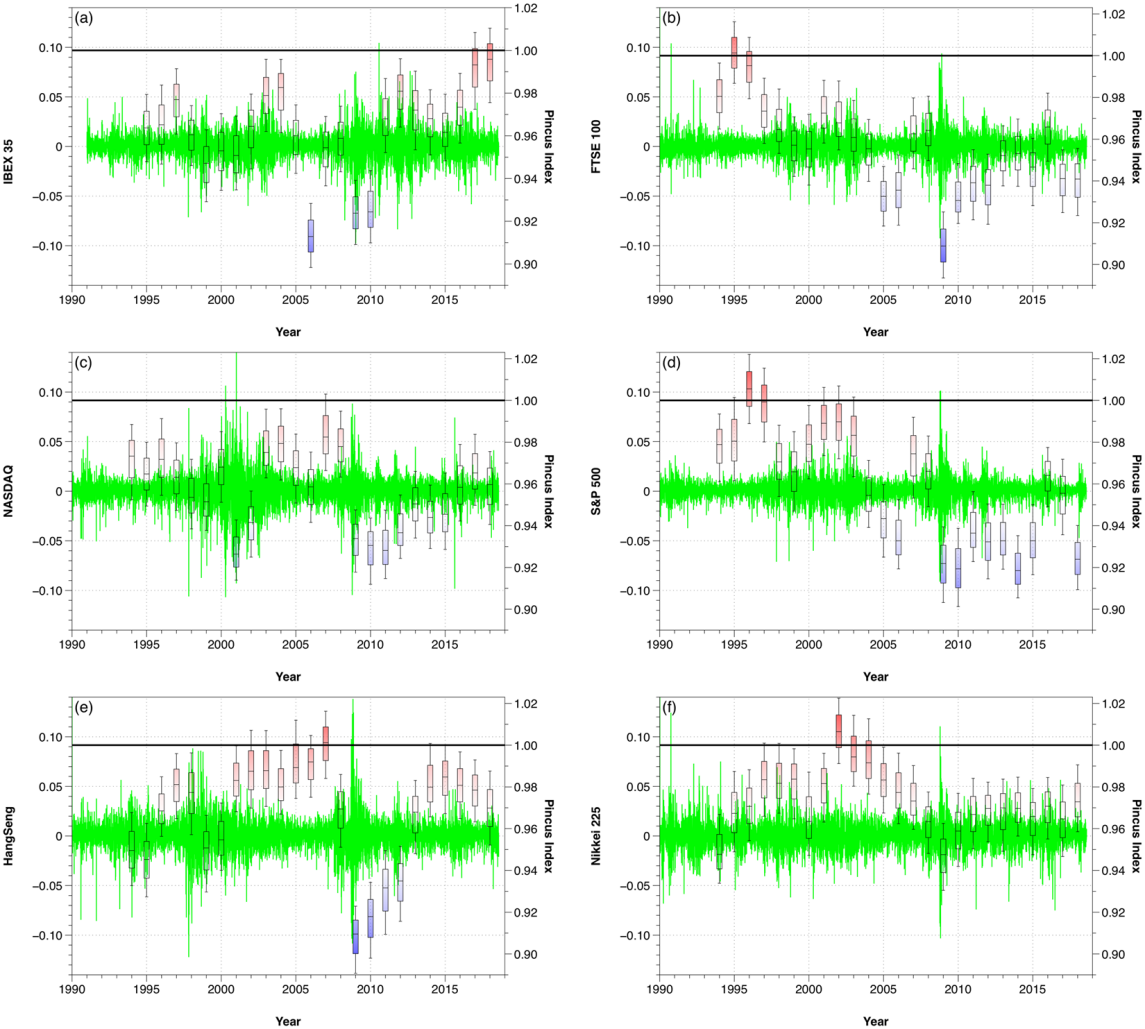
Evolution of the Pincus Index plotted over the log-ratio series considering information from the previous four years and rolling the window every year.

Figure 6 shows the same results for the different markets but plotted over the log-ratio transformed series. In general, we observe that high volatility events such as the 2008 crisis cause a decrease in the Pincus Index. At those moments, the stock markets tend to go down, creating downward patterns for several days and providing less randomness to the market because the behavior of the agents is more predictable. However, the influence of those events is different for each market. For example, in Fig. 6 we can see that after the 2008 crisis the Hang Seng evolved towards a high Pincus Index, while the S&P 500 stayed at low values ever since, showing differences in the preferences of the agents and their adaptation to the new conditions.

**Figure 7:**
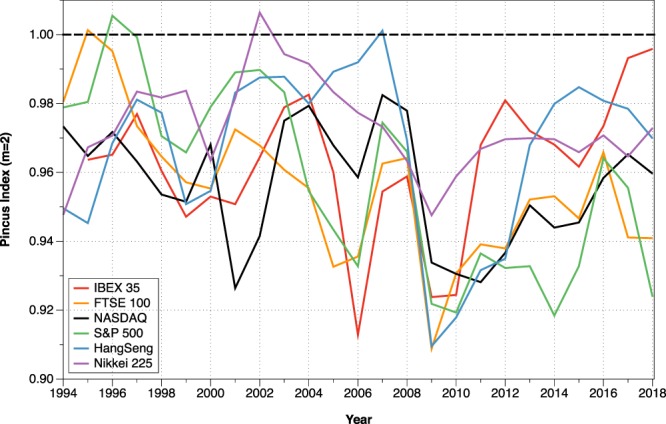
Comparison of the Pincus Index considering information from the previous four years and rolling the window every year.

As stated before, the Pincus Index provides a way to compare the randomness of different stock markets by merely comparing the values. In Fig. 7, we show the evolution of the PI in the last decades for the above-mentioned markets using four years of data. As the core calculation of the Pincus Index is based on counting patterns of data, it is foreseeable that a model created nowadays to predict the FTSE 100 or the S&P 500 would have greater success than one created for the IBEX 35.

In the Supplementary Information, we show similar calculations but using a different amount of data: considering two years of data and rolling the window every six months, one year rolling every three months, and six months rolling every month. In general, we observe that the level of randomness is dependent on the amount of data considered. We validate our results with a similar calculation of the Pincus Index using Sample Entropy instead of Approximate Entropy, but given the fact that Sample Entropy does not have a maximum value due to its construction, it becomes necessary to choose a value of *r* for those calculations.

The applicability of the Pincus Index is not limited to the stock markets or finance. In the Supplementary Information, we also show calculations for individual stocks (Google and Apple) as an example of its application.

## Discussion and Conclusions

In this paper, we have pursued two main objectives. The first of those was to provide a quantitative measure to determine the degree of randomness of evolving data series without being based on any underlying model or assuming any previous structure in the data series. The second one was to use this measure to evaluate the predictability of different stock markets.

To do so, we have analyzed Approximate Entropy and explained its main limitations for the analysis of financial data, i.e., the arbitrariness on the selection of *r* and the non-existence of a known alphabet. To solve those problems, we have proposed a methodology combining Monte Carlo simulations, the bootstrapping of the sequences and the selection of the MaxApEn value, defining an index of randomness useful for comparisons which is not limited to the stock markets.

From the results of the calculations, we observe that all the analyzed markets go through periods without patterns of behavior, and through other moments when the series are statistically different from total randomness. It highlights how different markets behaved similarly during the 2008 crisis but evolved differently after it. In general, we observe that the degree of predictability increases in times of crisis; in those moments, the market chains down sessions creating more patterns repeated by the agents. However, we do not observe that the regularity techniques studied in this paper can serve to predict an imminent “crash”. Thus, although previous research suggests that these algorithms can serve as leading indicators of the existence of crises or bubbles^4,8,10^, the results of this work cannot validate those conclusions. A situation such as the bubble of the *dot com* is not reflected in the NASDAQ before the index begins to fall, nor the falls in the Hang Seng in 1997–1998 can be predicted using ApEn or the Pincus Index. Nevertheless, some interesting behaviors stand out, such as the high predictability of the IBEX 35 in 2006 in the middle of a real estate bubble (Fig. 6), showing an “abnormal” situation given the general behavior of this index.

It is essential to notice that the proposed definition of the Pincus Index differs from the definition of {*m*, *N*}-random in three aspects^7^. First, the approach of using Monte Carlo simulations and bootstrapping is of practical use and can be more refined by increasing the number of repetitions, but we are in the field of statistics and not in the purest mathematics. Second, while {*m*, *N*}-random is defined as the maximum possible value of ApEn, we have defined the Pincus Index as a ratio to the median of the Monte Carlo simulations, not as a ratio to the maximum result of those simulations. As stated before, it implies that the Pincus Index can be greater than one, but we believe that using the 5%, 50% and 95% percentiles of the Monte Carlo simulations of MaxApEn is more representative. Finally, instead of using the euclidean difference to determine the distance between a sequence and the maximum randomness of its alphabet, we have defined the Pincus Index as a ratio, allowing comparisons between different alphabets.

Approximate Entropy is not the only measure of complexity used to characterize the markets. Other approaches such as the generalized Hurst exponent^20^, Kolmogorov complexity^21^ or LZ complexity^22^ have been applied to gain information on the stock markets which is different from the knowledge acquired by mainstream econometrics. Similarly, the entropy concept has been used in several of its conceptions to provide a different perspective from moment statistics or classical modeling of the stock markets^23–25^ and finances in general^26^. All those approaches provide different information and cannot be compared directly, albeit complementarily, they provide useful insights on the behavior of the markets. In this regard, Approximate Entropy deals with the measure of randomness, and, with the methodology presented in this paper, we have shown how the randomness of the stock markets changes through time, as suggested by the Adaptive Markets Hypothesis. The behavior of the agents in the market creates patterns which can be measured and quantified, and the Pincus Index can point to the modelers which markets have more repetitions at a specified time.

## Supplementary information

Supplementary Information

## Data Availability

The data have been obtained from Yahoo finances. For the IBEX 35, we only have data since 1991, and the data for the period 1991–1993 have been obtained from the site *infomercados*.*com* because they are not available in the Yahoo database. For the FTSE 100 index, the data have been obtained from the Wall Street Journal, and completed for the period 1990–1994 with data from the site *advfn*.*com*.
