# Supplementary material for: Quantifying the randomness of the stock markets
Source: Sci Rep. 2019 Sep 4;9:12761. doi: 10.1038/s41598-019-49320-9 (PMC6726611; doi:10.1038/s41598-019-49320-9)
Supplement: Supplementary file 1 — Supplementary Information [file 41598_2019_49320_MOESM1_ESM.pdf]

Quantifying the randomness of the stock markets Supplementary Material

Alfonso Delgado-Bonal[1]

[1]Universidad Nacional de Educación a Distancia, Facultad de Ciencias Económicas y Empresariales, España

alfonso.delgadobonal@nasa.gov

July 8, 2019

Contents

1 Statistical description of the data **1**

2 Pincus Index with different time frames plotted over market prices **8**
- 2.1 Spain - IBEX 35 8
- 2.2 UK - FTSE 100 9
- 2.3 USA - NASDAQ 10
- 2.4 USA - S&P 500 11
- 2.5 Hong Kong - Hang Seng 12
- 2.6 Japan - Nikkei 225 13

3 Pincus Index with different time frames plotted over log-ratio series **14**
- 3.1 Comparative evolution of the analyzed markets 18

4 Pincus Index for individual stocks **19**

5 Pincus Index using Sample Entropy **20**

1 Statistical description of the data

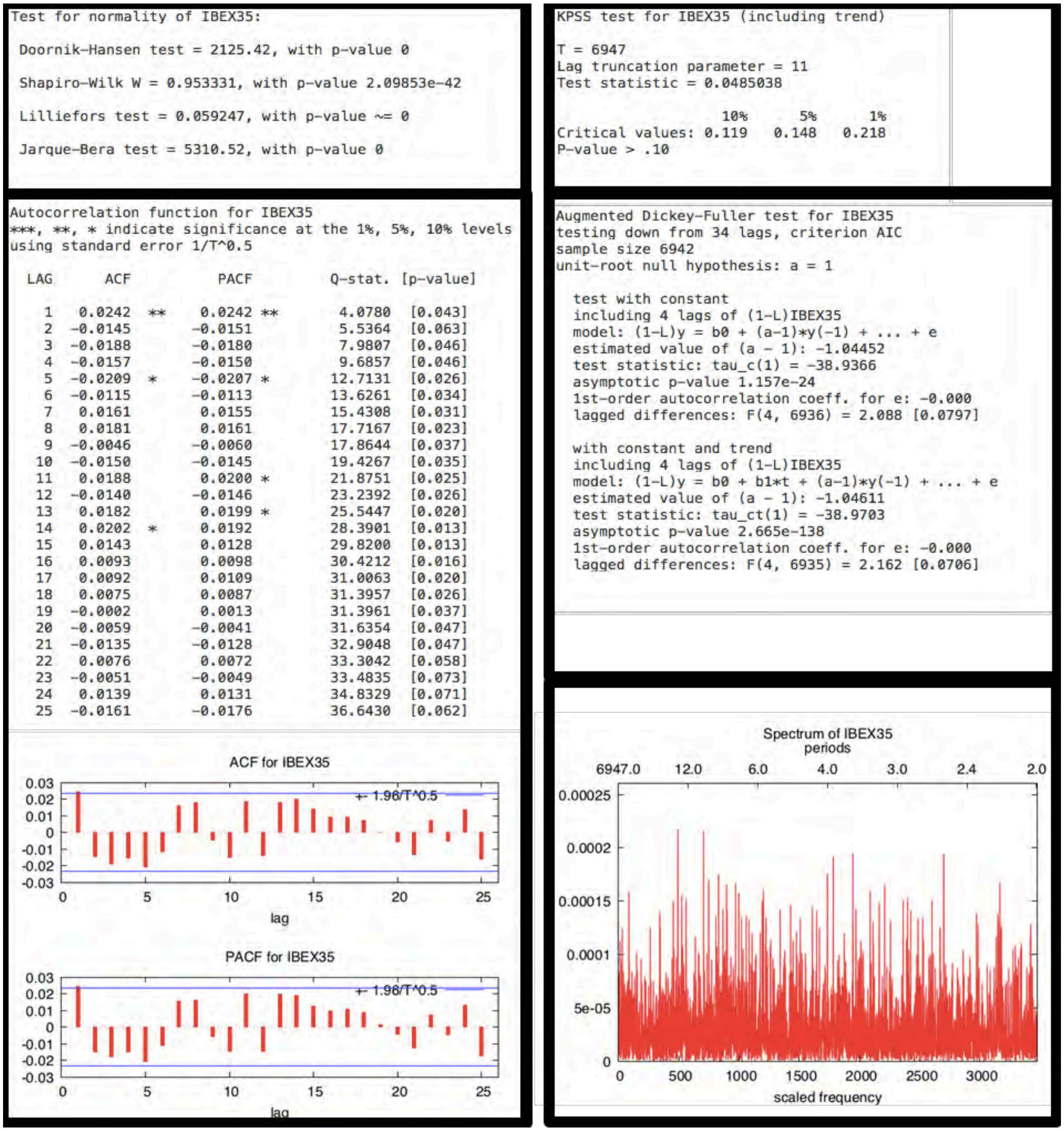

Test for normality of IBEX35:

Doornik-Hansen test = 2125.42, with p-value 0

Shapiro-Wilk W = 0.953331, with p-value 2.09853e-42

Lilliefors test = 0.059247, with p-value ~= 0

Jarque-Bera test = 5310.52, with p-value 0

KPSS test for IBEX35 (including trend)

T = 6947
Lag truncation parameter = 11
Test statistic = 0.0485038

	10%	5%	1%
Critical values:	0.119	0.148	0.218

P-value > .10

Autocorrelation function for IBEX35
***, **, * indicate significance at the 1%, 5%, 10% levels
using standard error 1/T^0.5

LAG	ACF		PACF		Q-stat.	[p-value]
1	0.0242	**	0.0242	**	4.0780	[0.043]
2	-0.0145		-0.0151		5.5364	[0.063]
3	-0.0188		-0.0180		7.9807	[0.046]
4	-0.0157		-0.0150		9.6857	[0.046]
5	-0.0209	*	-0.0207	*	12.7131	[0.026]
6	-0.0115		-0.0113		13.6261	[0.034]
7	0.0161		0.0155		15.4308	[0.031]
8	0.0181		0.0161		17.7167	[0.023]
9	-0.0046		-0.0060		17.8644	[0.037]
10	-0.0150		-0.0145		19.4267	[0.035]
11	0.0188		0.0200	*	21.8751	[0.025]
12	-0.0140		-0.0146		23.2392	[0.026]
13	0.0182		0.0199	*	25.5447	[0.020]
14	0.0202	*	0.0192		28.3901	[0.013]
15	0.0143		0.0128		29.8200	[0.013]
16	0.0093		0.0098		30.4212	[0.016]
17	0.0092		0.0109		31.0063	[0.020]
18	0.0075		0.0087		31.3957	[0.026]
19	-0.0002		0.0013		31.3961	[0.037]
20	-0.0059		-0.0041		31.6354	[0.047]
21	-0.0135		-0.0128		32.9048	[0.047]
22	0.0076		0.0072		33.3042	[0.058]
23	-0.0051		-0.0049		33.4835	[0.073]
24	0.0139		0.0131		34.8329	[0.071]
25	-0.0161		-0.0176		36.6430	[0.062]

Augmented Dickey-Fuller test for IBEX35
testing down from 34 lags, criterion AIC
sample size 6942
unit-root null hypothesis: a = 1

test with constant
including 4 lags of (1-L)IBEX35
model: (1-L)y = b0 + (a-1)*y(-1) + ... + e
estimated value of (a - 1): -1.04452
test statistic: tau_c(1) = -38.9366
asymptotic p-value 1.157e-24
1st-order autocorrelation coeff. for e: -0.000
lagged differences: F(4, 6936) = 2.088 [0.0797]

with constant and trend
including 4 lags of (1-L)IBEX35
model: (1-L)y = b0 + b1*t + (a-1)*y(-1) + ... + e
estimated value of (a - 1): -1.04611
test statistic: tau_ct(1) = -38.9703
asymptotic p-value 2.665e-138
1st-order autocorrelation coeff. for e: -0.000
lagged differences: F(4, 6935) = 2.162 [0.0706]

Figure 1: Statistical characterization of the IBEX 35 dataset.

```
Test for normality of v1:

 Doornik-Hansen test = 4650.06, with p-value 0

 Shapiro-Wilk W = 0.932658, with p-value 5.11908e-49

 Lilliefors test = 0.0674379, with p-value ~= 0

 Jarque-Bera test = 14909.6, with p-value 0
```

```
KPSS test for v1 (including trend)

T = 7205
Lag truncation parameter = 11
Test statistic = 0.0480165

                   10%      5%      1%
Critical values: 0.119   0.148   0.218
P-value > .10
```

```
Autocorrelation function for v1
***, **, * indicate significance at the 1%, 5%, 10% levels
using standard error 1/T^0.5

  LAG      ACF          PACF         Q-stat. [p-value]

    1  -0.0429  ***  -0.0429 ***    13.2358  [0.000]
    2  -0.0362  ***  -0.0381 ***    22.7015  [0.000]
    3  -0.0450  ***  -0.0484 ***    37.3066  [0.000]
    4   0.0343  ***   0.0289 **     45.8059  [0.000]
    5  -0.0420  ***  -0.0430 ***    58.5301  [0.000]
    6  -0.0300  **   -0.0338 ***    65.0286  [0.000]
    7  -0.0040       -0.0073        65.1432  [0.000]
    8   0.0319  ***   0.0242 **     72.4976  [0.000]
    9   0.0053        0.0071        72.7026  [0.000]
   10  -0.0098       -0.0076        73.4004  [0.000]
   11   0.0016        0.0016        73.4195  [0.000]
   12  -0.0076       -0.0107        73.8397  [0.000]
   13   0.0114        0.0118        74.7846  [0.000]
   14  -0.0180       -0.0149        77.1366  [0.000]
   15   0.0026        0.0010        77.1854  [0.000]
   16   0.0051        0.0045        77.3726  [0.000]
   17  -0.0087       -0.0116        77.9228  [0.000]
   18  -0.0233  **   -0.0221 *      81.8596  [0.000]
   19  -0.0144       -0.0179        83.3618  [0.000]
   20   0.0101        0.0054        84.1006  [0.000]
   21   0.0032        0.0010        84.1763  [0.000]
   22   0.0088        0.0096        84.7419  [0.000]
   23  -0.0160       -0.0158        86.5862  [0.000]
   24  -0.0077       -0.0123        87.0103  [0.000]
   25   0.0156        0.0147        88.7690  [0.000]
```

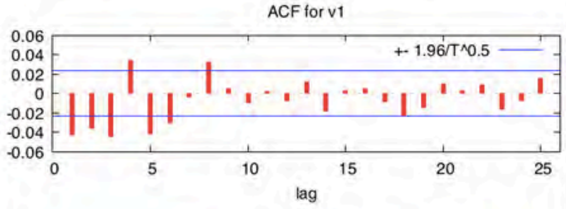

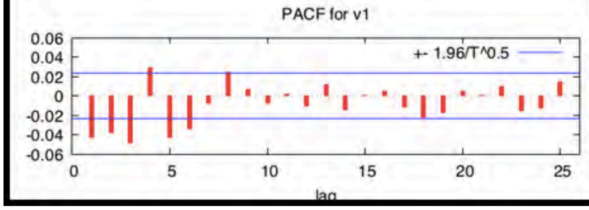


```
Augmented Dickey-Fuller test for v1
testing down from 34 lags, criterion AIC
sample size 7171
unit-root null hypothesis: a = 1

  test with constant
  including 33 lags of (1-L)v1
  model: (1-L)y = b0 + (a-1)*y(-1) + ... + e
  estimated value of (a - 1): -1.29143
  test statistic: tau_c(1) = -15.8466
  asymptotic p-value 1.264e-37
  1st-order autocorrelation coeff. for e: -0.000
  lagged differences: F(33, 7136) = 3.468 [0.0000]

  with constant and trend
  including 33 lags of (1-L)v1
  model: (1-L)y = b0 + b1*t + (a-1)*y(-1) + ... + e
  estimated value of (a - 1): -1.29318
  test statistic: tau_ct(1) = -15.8573
  asymptotic p-value 1.98e-47
  1st-order autocorrelation coeff. for e: -0.000
  lagged differences: F(33, 7135) = 3.471 [0.0000]
```

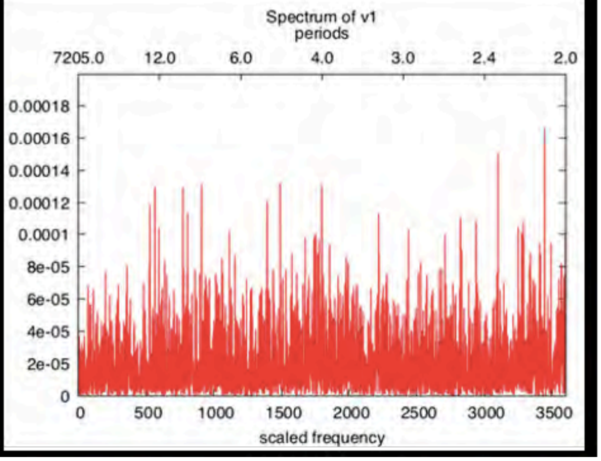

Figure 2: Statistical characterization of the FTSE 100 dataset.

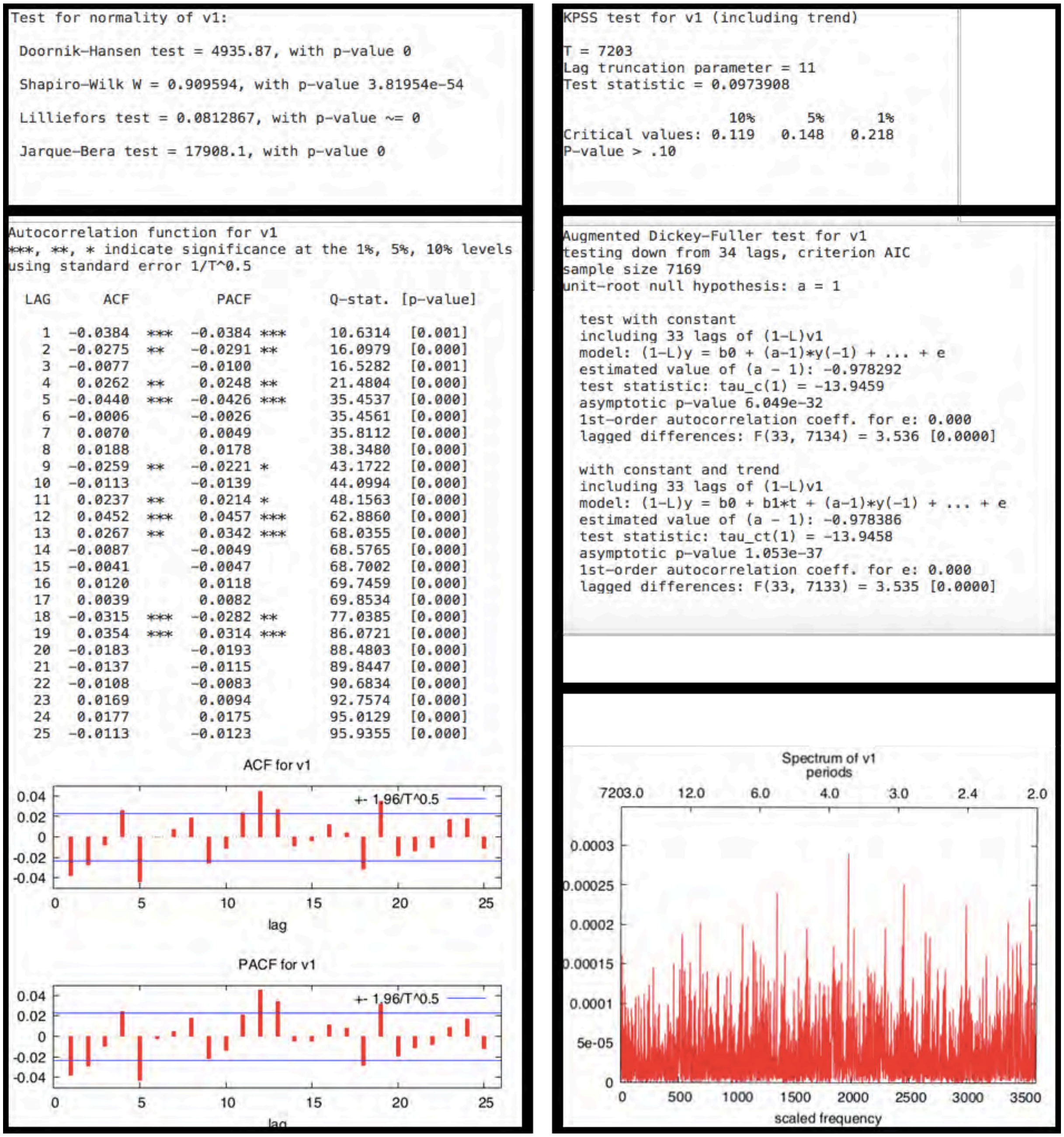

Figure 3: Statistical characterization of the NASDAQ dataset.

```
Test for normality of SandP500:

 Doornik-Hansen test = 5660.39, with p-value 0

 Shapiro-Wilk W = 0.917796, with p-value 1.8539e-52

 Lilliefors test = 0.085372, with p-value ~= 0

 Jarque-Bera test = 21706.2, with p-value 0
```

```
KPSS test for SandP500 (including trend)

T = 7203
Lag truncation parameter = 11
Test statistic = 0.103116

                   10%      5%      1%
Critical values: 0.119   0.148   0.218
P-value > .10
```

```
Autocorrelation function for SandP500
***, **, * indicate significance at the 1%, 5%, 10% levels
using standard error 1/T^0.5

  LAG      ACF           PACF          Q-stat.  [p-value]

    1  -0.0264  **   -0.0264 **      5.0264   [0.025]
    2  -0.0458  ***  -0.0466 ***    20.1598   [0.000]
    3   0.0021       -0.0004        20.1909   [0.000]
    4  -0.0081       -0.0102        20.6654   [0.000]
    5  -0.0413  ***  -0.0419 ***    32.9913   [0.000]
    6   0.0020       -0.0011        33.0212   [0.000]
    7  -0.0369  ***  -0.0409 ***    42.8333   [0.000]
    8   0.0100        0.0078        43.5511   [0.000]
    9  -0.0025       -0.0065        43.5951   [0.000]
   10   0.0186        0.0175        46.0847   [0.000]
   11  -0.0121       -0.0124        47.1464   [0.000]
   12   0.0408  ***   0.0390 ***    59.1646   [0.000]
   13   0.0154        0.0172        60.8769   [0.000]
   14  -0.0151       -0.0119        62.5222   [0.000]
   15  -0.0347  ***  -0.0321 ***    71.2338   [0.000]
   16   0.0314  ***   0.0280 **     78.3542   [0.000]
   17   0.0002        0.0038        78.3547   [0.000]
   18  -0.0359  ***  -0.0335 ***    87.6423   [0.000]
   19   0.0083        0.0083        88.1398   [0.000]
   20   0.0128        0.0081        89.3295   [0.000]
   21  -0.0277  **   -0.0248 **     94.8916   [0.000]
   22   0.0074        0.0026        95.2823   [0.000]
   23   0.0037        0.0021        95.3835   [0.000]
   24   0.0035        0.0036        95.4737   [0.000]
   25  -0.0181       -0.0205 *      97.8505   [0.000]
```

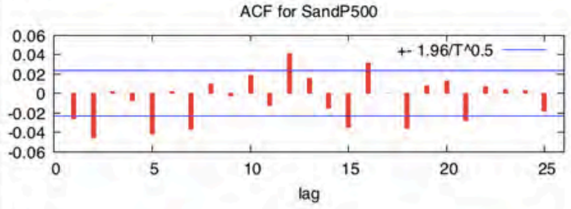

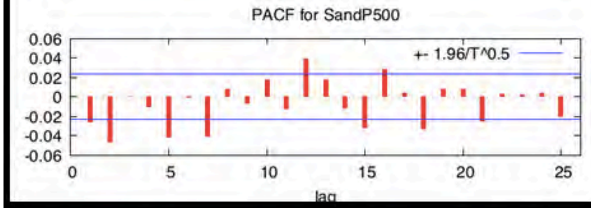


```
Augmented Dickey-Fuller test for SandP500
testing down from 34 lags, criterion AIC
sample size 7169
unit-root null hypothesis: a = 1

  test with constant
  including 33 lags of (1-L)SandP500
  model: (1-L)y = b0 + (a-1)*y(-1) + ... + e
  estimated value of (a - 1): -1.1739
  test statistic: tau_c(1) = -15.0596
  asymptotic p-value 2.43e-35
  1st-order autocorrelation coeff. for e: 0.000
  lagged differences: F(33, 7134) = 3.835 [0.0000]

  with constant and trend
  including 33 lags of (1-L)SandP500
  model: (1-L)y = b0 + b1*t + (a-1)*y(-1) + ... + e
  estimated value of (a - 1): -1.17438
  test statistic: tau_ct(1) = -15.0618
  asymptotic p-value 2.512e-43
  1st-order autocorrelation coeff. for e: 0.000
  lagged differences: F(33, 7133) = 3.835 [0.0000]
```

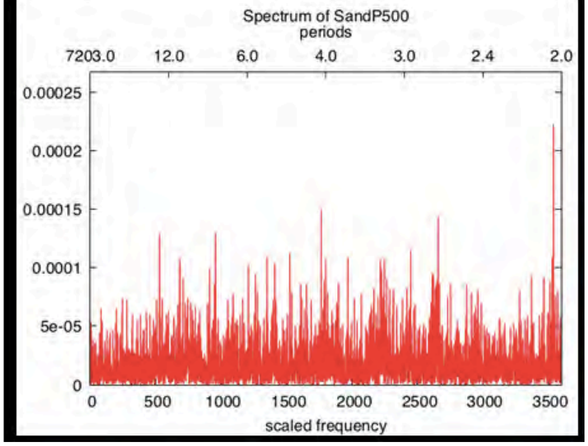

Figure 4: Statistical characterization of the S&P 500 dataset.

```
Test for normality of HangSeng:

 Doornik-Hansen test = 4572.23, with p-value 0

 Shapiro-Wilk W = 0.927881, with p-value 7.77872e-50

 Lilliefors test = 0.0729393, with p-value ~= 0

 Jarque-Bera test = 15105.1, with p-value 0
```

```
KPSS test for HangSeng (including trend)

T = 7064
Lag truncation parameter = 11
Test statistic = 0.0445898

                   10%      5%      1%
Critical values: 0.119   0.148   0.218
P-value > .10
```

```
Autocorrelation function for HangSeng
***, **, * indicate significance at the 1%, 5%, 10% levels
using standard error 1/T^0.5

  LAG      ACF           PACF          Q-stat. [p-value]

    1   0.0350  ***   0.0350 ***      8.6339  [0.003]
    2  -0.0143        -0.0156        10.0865  [0.006]
    3   0.0259  **    0.0270 **      14.8386  [0.002]
    4  -0.0322  ***  -0.0344 ***     22.1629  [0.000]
    5  -0.0423  ***  -0.0392 ***     34.8366  [0.000]
    6   0.0264  **    0.0277 **      39.7531  [0.000]
    7  -0.0305  **   -0.0322 ***     46.3441  [0.000]
    8   0.0375  ***   0.0420 ***     56.2861  [0.000]
    9  -0.0197  *    -0.0280 **      59.0378  [0.000]
   10   0.0084        0.0134         59.5420  [0.000]
   11   0.0186        0.0153         61.9935  [0.000]
   12   0.0147        0.0141         63.5185  [0.000]
   13   0.0060        0.0086         63.7704  [0.000]
   14   0.0254  **    0.0199 *       68.3527  [0.000]
   15  -0.0051       -0.0015         68.5337  [0.000]
   16  -0.0075       -0.0083         68.9278  [0.000]
   17  -0.0141       -0.0116         70.3276  [0.000]
   18  -0.0085       -0.0068         70.8396  [0.000]
   19  -0.0013        0.0007         70.8513  [0.000]
   20   0.0329  ***   0.0316 ***     78.5435  [0.000]
   21   0.0098        0.0077         79.2200  [0.000]
   22  -0.0326  ***  -0.0359 ***     86.7382  [0.000]
   23  -0.0032       -0.0017         86.8092  [0.000]
   24  -0.0267  **   -0.0275 **      91.8810  [0.000]
   25   0.0175        0.0245 **      94.0563  [0.000]
```

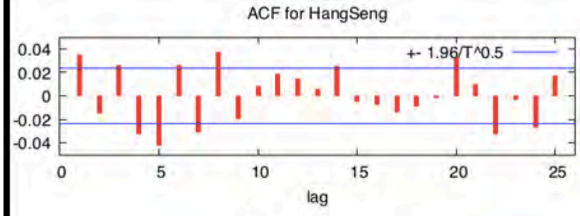

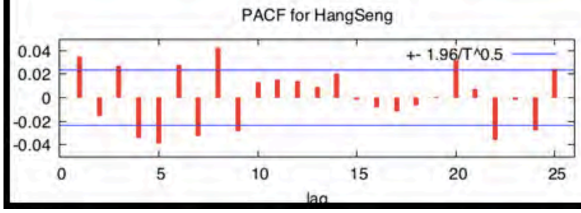


```
Augmented Dickey-Fuller test for HangSeng
testing down from 34 lags, criterion AIC
sample size 7039
unit-root null hypothesis: a = 1

  test with constant
  including 24 lags of (1-L)HangSeng
  model: (1-L)y = b0 + (a-1)*y(-1) + ... + e
  estimated value of (a - 1): -0.968144
  test statistic: tau_c(1) = -16.6689
  asymptotic p-value 6.91e-40
  1st-order autocorrelation coeff. for e: -0.000
  lagged differences: F(24, 7013) = 3.870 [0.0000]

  with constant and trend
  including 24 lags of (1-L)HangSeng
  model: (1-L)y = b0 + b1*t + (a-1)*y(-1) + ... + e
  estimated value of (a - 1): -0.972213
  test statistic: tau_ct(1) = -16.7055
  asymptotic p-value 7.171e-52
  1st-order autocorrelation coeff. for e: -0.000
  lagged differences: F(24, 7012) = 3.871 [0.0000]
```

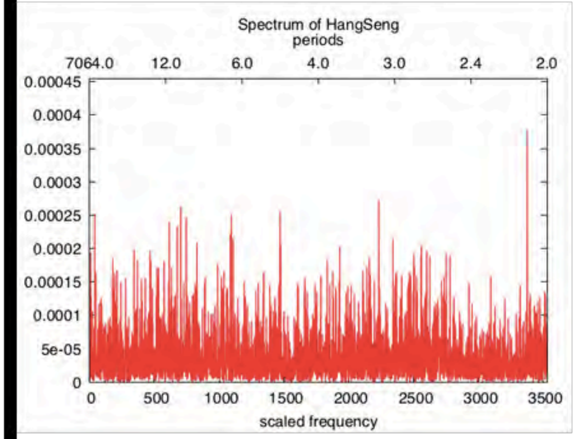

Figure 5: Statistical characterization of the Hang Seng dataset.

```
Test for normality of Nikkei225:

 Doornik-Hansen test = 2542.25, with p-value 0

 Shapiro-Wilk W = 0.962558, with p-value 3.34171e-39

 Lilliefors test = 0.0445823, with p-value ~= 0

 Jarque-Bera test = 6213.36, with p-value 0
```

```
KPSS test for Nikkei225 (including trend)

T = 7033
Lag truncation parameter = 11
Test statistic = 0.0390295

                  10%      5%      1%
Critical values: 0.119   0.148   0.218
P-value > .10
```

```
Autocorrelation function for Nikkei225
***, **, * indicate significance at the 1%, 5%, 10% levels
using standard error 1/T^0.5

  LAG      ACF          PACF         Q-stat. [p-value]

    1   0.0482  ***   0.0482 ***    16.3486  [0.000]
    2  -0.0375  ***  -0.0399 ***    26.2229  [0.000]
    3  -0.0064       -0.0026        26.5127  [0.000]
    4  -0.0106       -0.0116        27.2966  [0.000]
    5   0.0005        0.0013        27.2985  [0.000]
    6  -0.0248  **   -0.0259 **     31.6300  [0.000]
    7   0.0007        0.0032        31.6332  [0.000]
    8   0.0082        0.0059        32.1030  [0.000]
    9   0.0192        0.0185        34.6905  [0.000]
   10   0.0234  **    0.0216 *      38.5583  [0.000]
   11   0.0107        0.0101        39.3615  [0.000]
   12  -0.0026       -0.0022        39.4094  [0.000]
   13  -0.0174       -0.0158        41.5543  [0.000]
   14  -0.0008        0.0015        41.5591  [0.000]
   15   0.0136        0.0134        42.8671  [0.000]
   16  -0.0054       -0.0060        43.0727  [0.000]
   17   0.0084        0.0099        43.5750  [0.000]
   18   0.0130        0.0111        44.7656  [0.000]
   19   0.0075        0.0056        45.1655  [0.001]
   20  -0.0098       -0.0105        45.8495  [0.001]
   21  -0.0384  ***  -0.0362 ***    56.2374  [0.000]
   22  -0.0182       -0.0149        58.5637  [0.000]
   23  -0.0044       -0.0047        58.7034  [0.000]
   24   0.0262  **    0.0256 **     63.5583  [0.000]
   25   0.0232  *     0.0194        67.3728  [0.000]
```

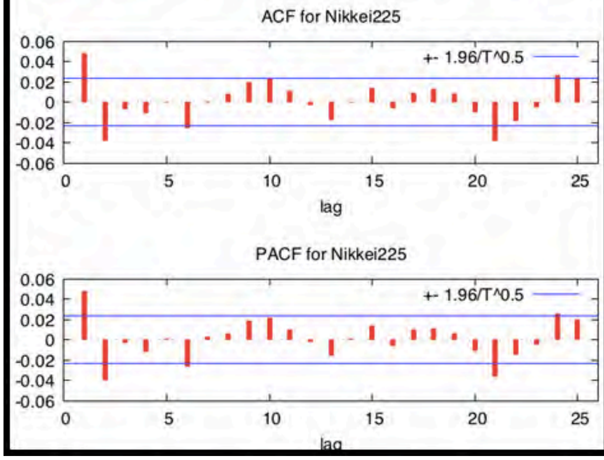


```
Augmented Dickey-Fuller test for Nikkei225
testing down from 34 lags, criterion AIC
sample size 7031
unit-root null hypothesis: a = 1

  test with constant
  including one lag of (1-L)Nikkei225
  model: (1-L)y = b0 + (a-1)*y(-1) + ... + e
  estimated value of (a - 1): -0.989749
  test statistic: tau_c(1) = -60.1863
  asymptotic p-value 0.0001
  1st-order autocorrelation coeff. for e: -0.000

  with constant and trend
  including one lag of (1-L)Nikkei225
  model: (1-L)y = b0 + b1*t + (a-1)*y(-1) + ... + e
  estimated value of (a - 1): -0.990819
  test statistic: tau_ct(1) = -60.2314
  asymptotic p-value 9.351e-87
  1st-order autocorrelation coeff. for e: -0.000
```

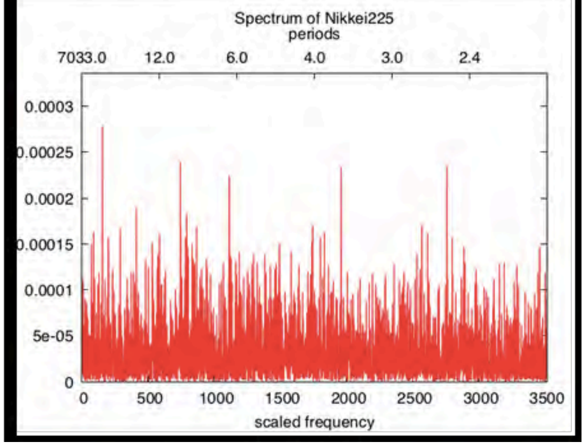

Figure 6: Statistical characterization of the Nikkei 225 dataset.

2 Pincus Index with different time frames plotted over market prices

We calculate the PI considering different time frames: four years of data moving the window every year, two years of data moving every six months, one year moving every three months, and six months moving every month. For four and two years situations we have represented the PI together with the 5% and 95% percentiles, while for the one year and six months situations we have only represented the PI with respect to the median. The only reason is that the number of points in the graph is substantial and if we plot all of them the behavior is unobservable.

2.1 Spain - IBEX 35

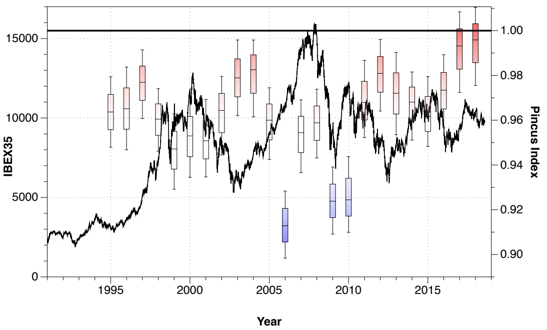

(a) Pincus index considering information from the previous four years and moving every year.

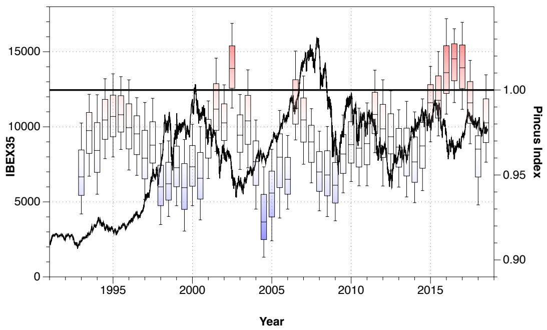

(b) Pincus index considering information from the previous two years and moving every six months.

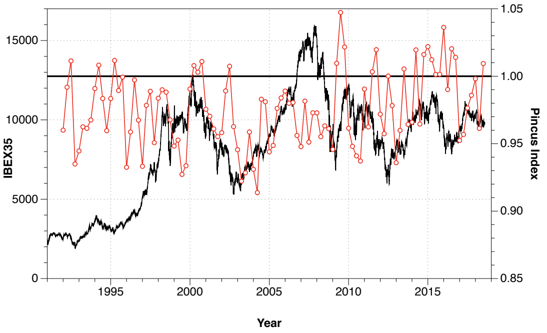

(c) Pincus index considering information from a previous year and moving every three months.

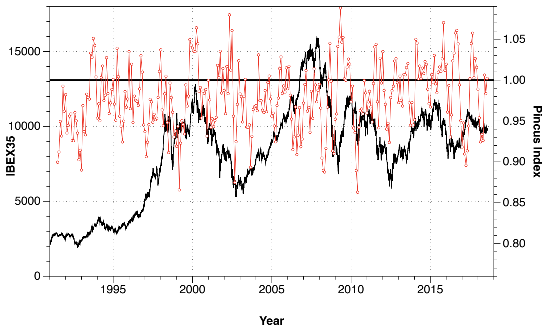

(d) Pincus index considering information from the previous six months and moving every month.

Figure 7: Evolution of the Pincus index for the IBEX 35 considering different number of points in the data series.

2.2 UK - FTSE 100

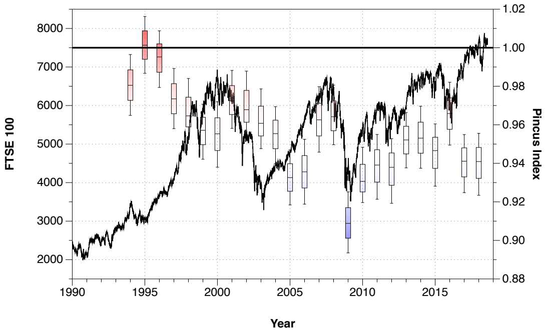

(a) Pincus index considering information from the previous four years and moving every year.

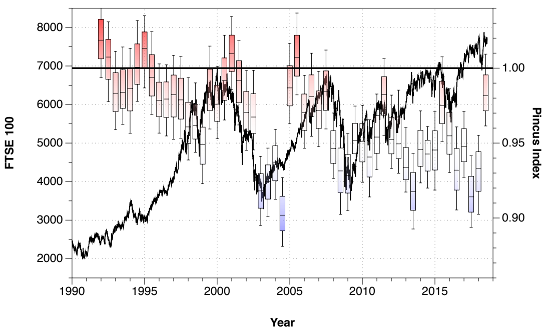

(b) Pincus index considering information from the previous two years and moving every six months.

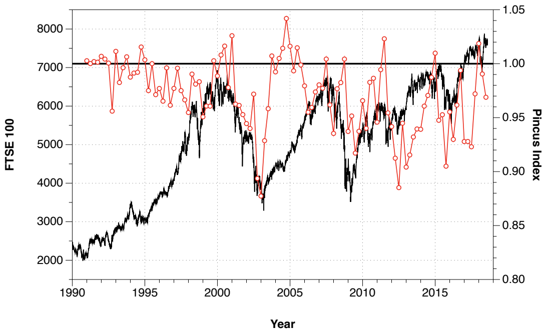

(c) Pincus index considering information from a previous year and moving every three months.

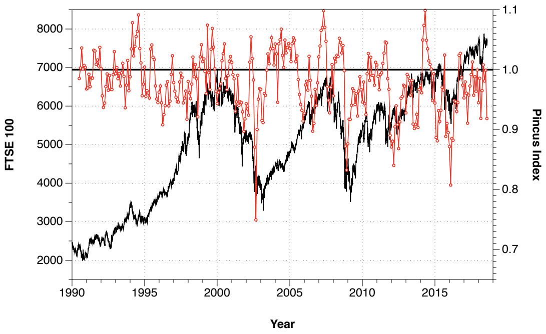

(d) Pincus index considering information from the previous six months and moving every month.

Figure 8: Evolution of the Pincus index for the FTSE 100 considering different number of points in the data series.

2.3 USA - NASDAQ

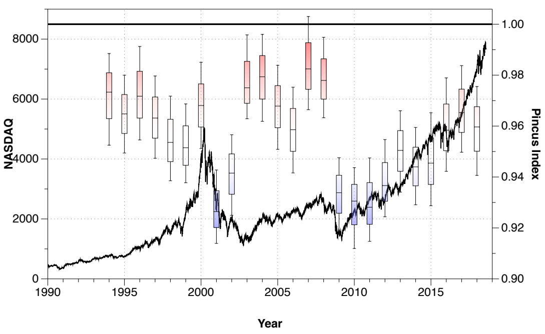

(a) Pincus index considering information from the previous four years and moving every year.

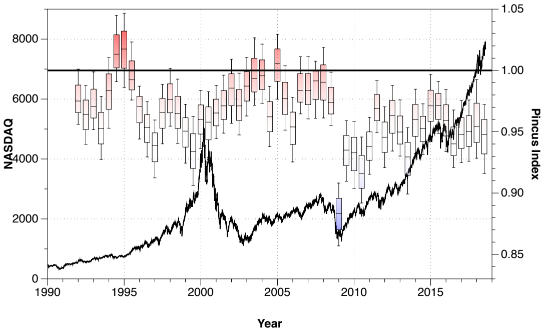

(b) Pincus index considering information from the previous two years and moving every six months.

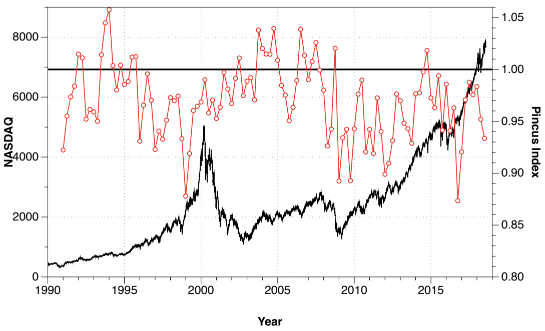

(c) Pincus index considering information from a previous year and moving every three months.

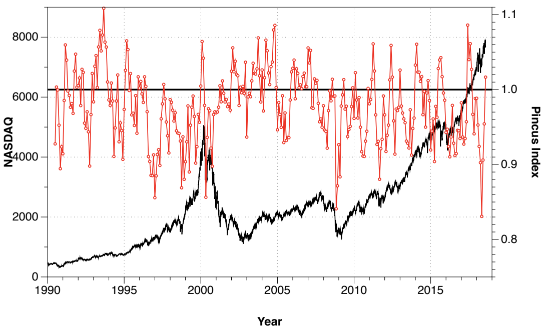

(d) Pincus index considering information from the previous six months and moving every month.

Figure 9: Evolution of the Pincus index for the NASDAQ considering different number of points in the data series.

2.4 USA - S&P 500

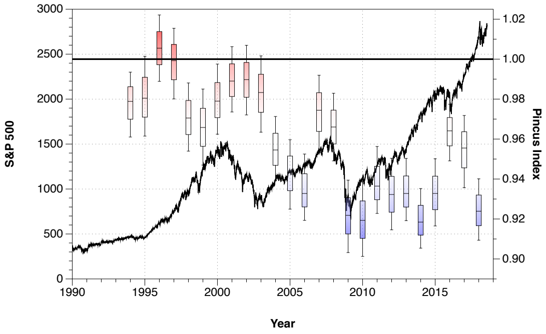

(a) Pincus index considering information from the previous four years and moving every year.

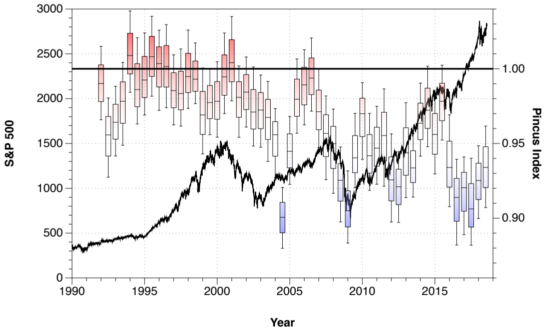

(b) Pincus index considering information from the previous two years and moving every six months.

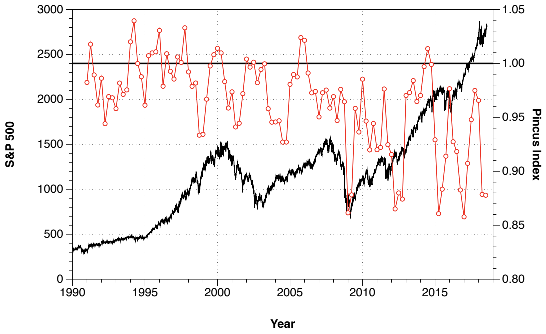

(c) Pincus index considering information from a previous year and moving every three months.

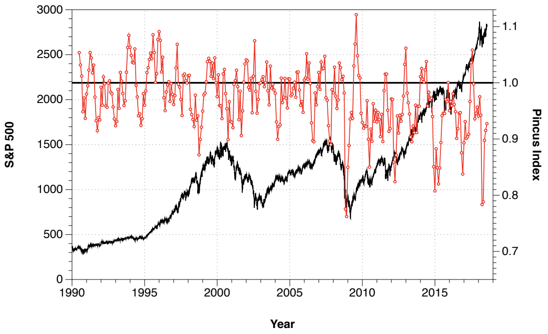

(d) Pincus index considering information from the previous six months and moving every month.

Figure 10: Evolution of the Pincus index for the S&P 500 considering different number of points in the data series.

2.5 Hong Kong - Hang Seng

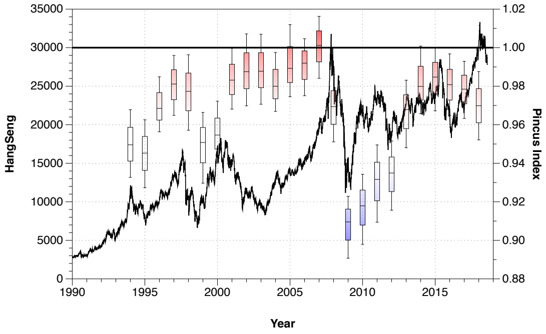

(a) Pincus index considering information from the previous four years and moving every year.

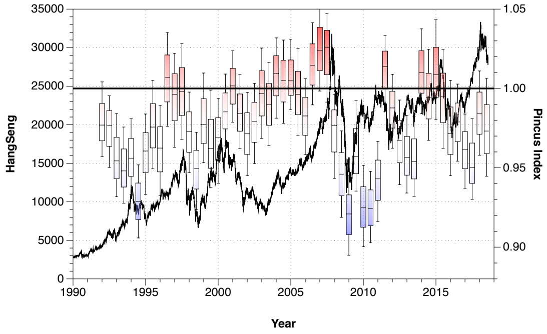

(b) Pincus index considering information from the previous two years and moving every six months.

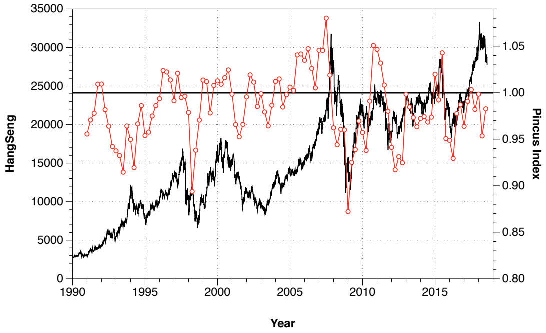

(c) Pincus index considering information from a previous year and moving every three months.

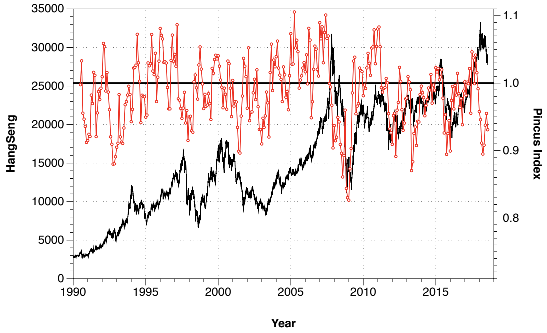

(d) Pincus index considering information from the previous six months and moving every month.

Figure 11: Evolution of the Pincus index for the Hang Seng considering different number of points in the data series.

2.6 Japan - Nikkei 225

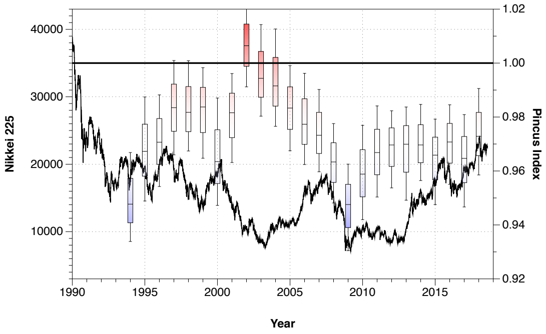

(a) Pincus index considering information from the previous four years and moving every year.

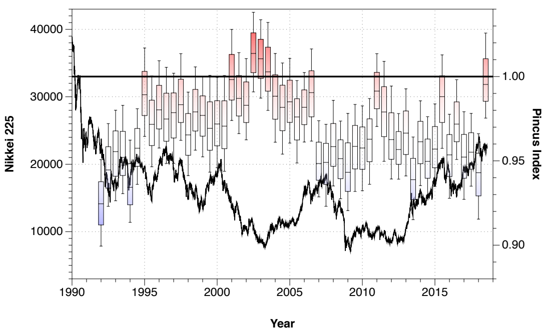

(b) Pincus index considering information from the previous two years and moving every six months.

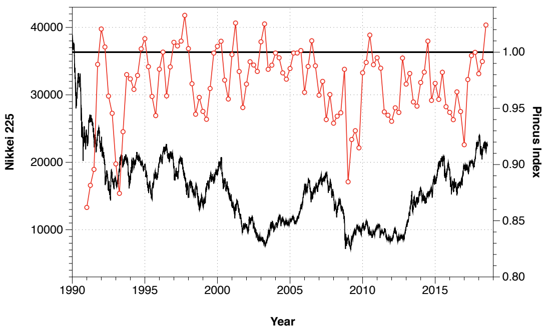

(c) Pincus index considering information from a previous year and moving every three months.

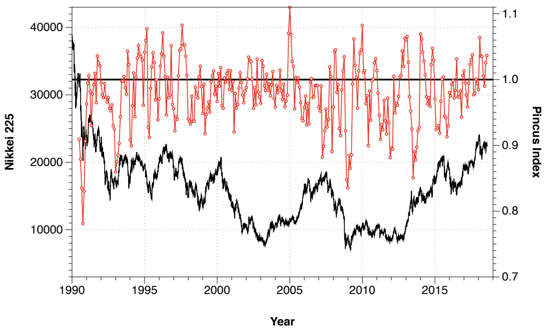

(d) Pincus index considering information from the previous six months and moving every month.

Figure 12: Evolution of the Pincus index for the Nikkei 225 considering different number of points in the data series.

3 Pincus Index with different time frames plotted over log-ratio series

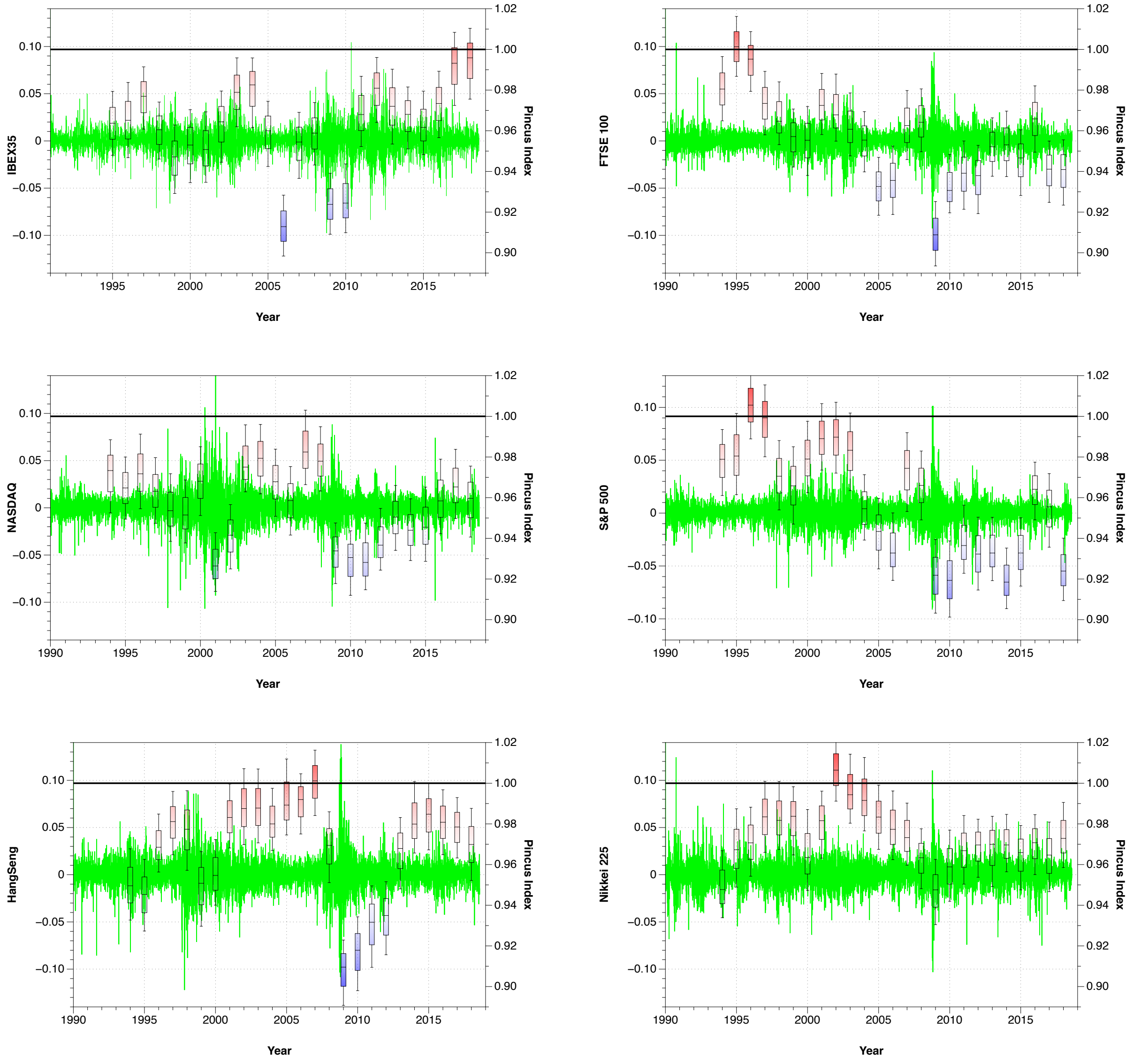

Figure 13: Evolution of the Pincus index on the log-ratio series considering information from the previous four years and moving every year.

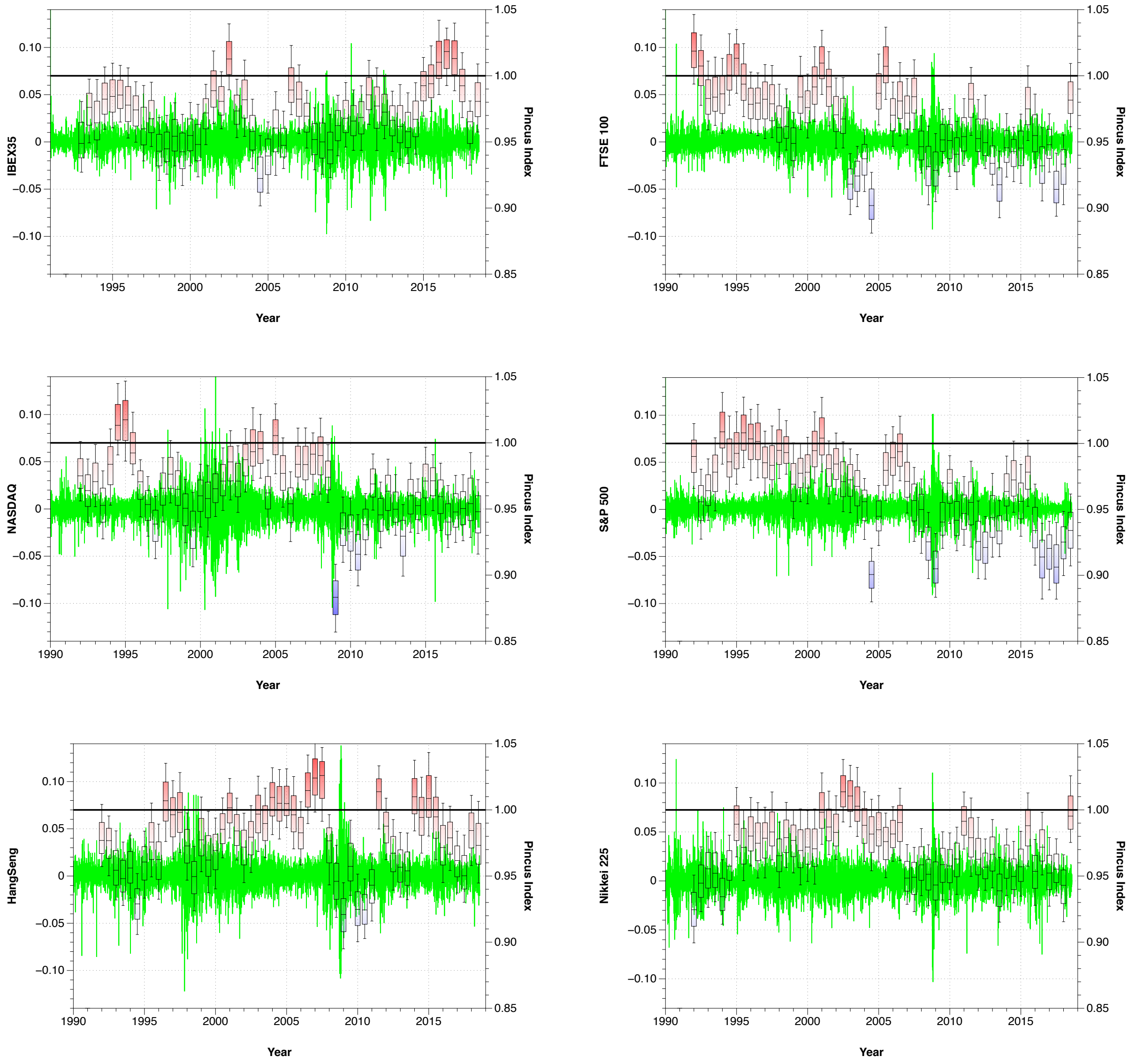

Figure 14: Evolution of the Pincus index on the log-ratio series considering information from the previous two years and moving every six months.

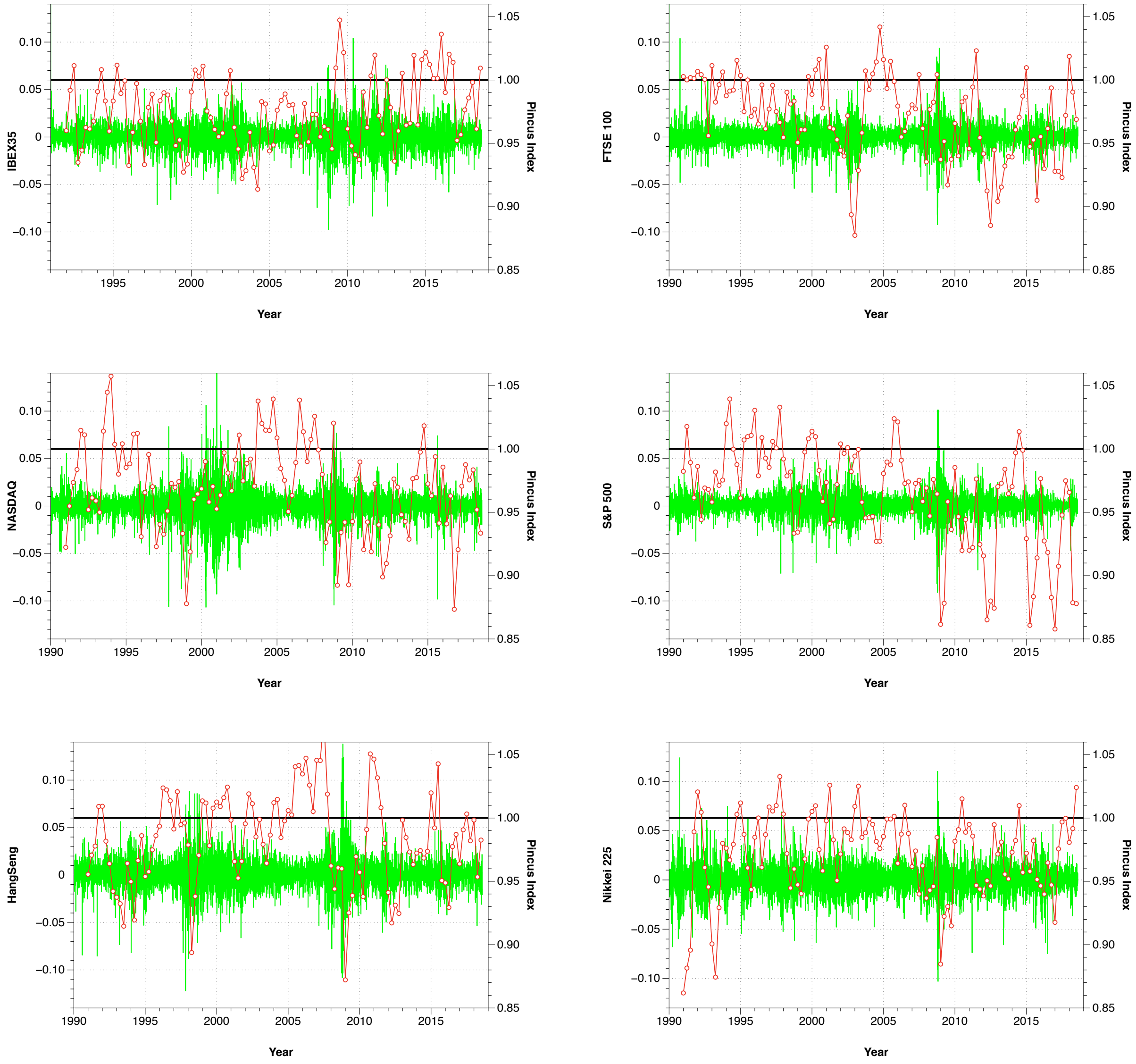

Figure 15: Evolution of the Pincus index on the log-ratio series considering information from the previous year and moving every three months.

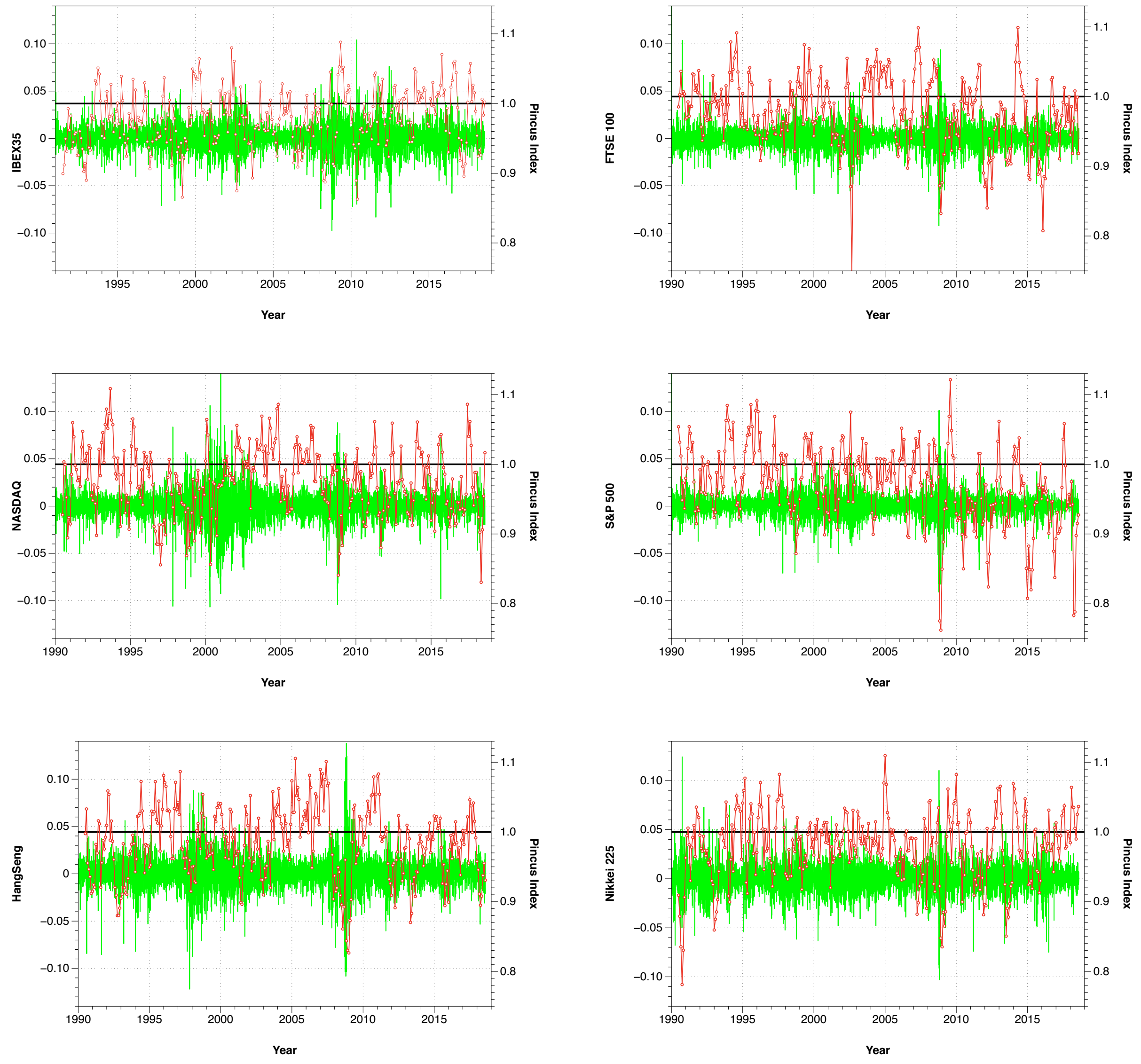

Figure 16: Evolution of the Pincus index on the log-ratio series considering information from the previous six months and moving every month.

3.1 Comparative evolution of the analyzed markets

In Figure 17 we summarize the results and show all the markets at different time frames.

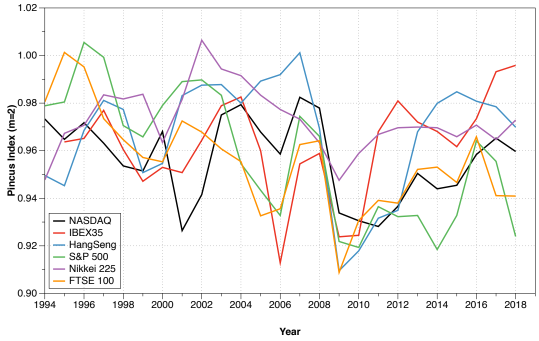

(a) Pincus index considering information from the previous four years and moving every year.

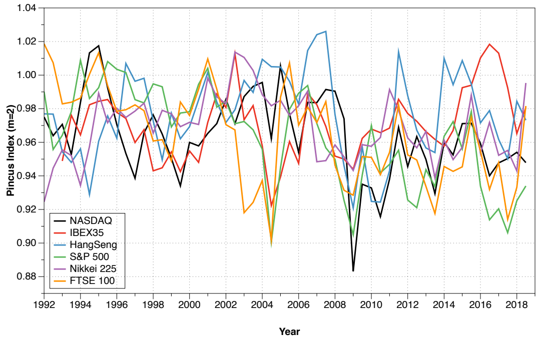

(b) Pincus index considering information from the previous two years and moving every six months.

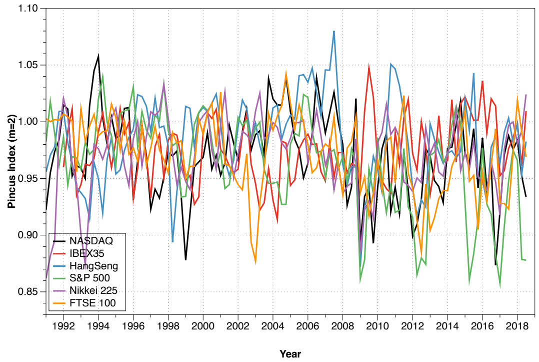

(c) Pincus index considering information from a previous year and moving every three months.

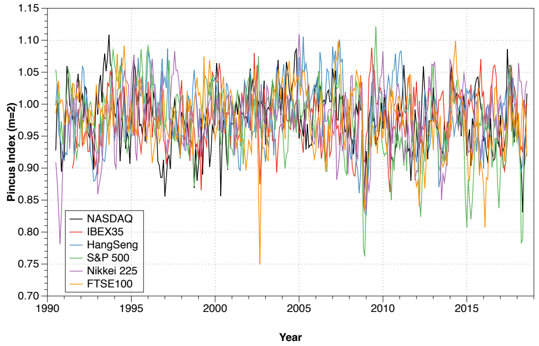

(d) Pincus index considering information from the previous six months and moving every month.

Figure 17: Evolution of the Pincus index for the different markets analyzed considering different number of points in the data series.

4 Pincus Index for individual stocks

As an example of its application, we show here the Pincus Index of two individual stocks over their price and over the log-ratio series.

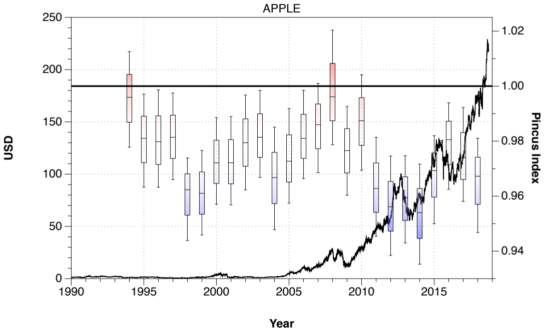

(a) Pincus index for Apple considering information from the previous four years and moving every year.

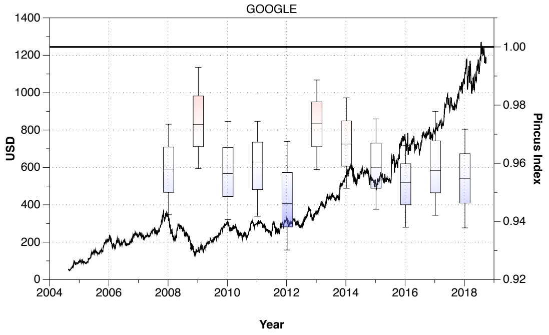

(b) Pincus index for Google considering information from the previous four years and moving every year.

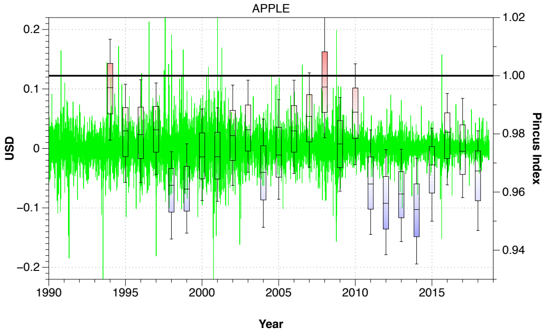

(c) Pincus index for Apple over its log-ratio series considering information from the previous four years and moving every year.

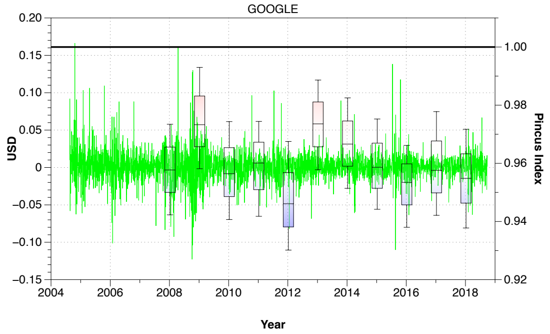

(d) Pincus index for Google over its log-ratio series considering information from the previous four years and moving every year.

Figure 18: Evolution of the Pincus index for two individual stocks.

5 Pincus Index using Sample Entropy

As in the previous section, we can evaluate the Pincus index of SampEn for different stock markets. However, due to the shape of SampEn, it is necessary to chose a value for r; in the following calculations we select $r = 0.2$. We show the results for the different markets depending on the size of the window. For example, Figure 19 presents the results for the six analyzed markets (IBEX35, FTSE 100, NASDAQ, S&P 500, Hang Seng, Nikkei 225) using four-year windows and moving them every year. The value corresponding to 1994 is the first one obtained, knowing the data from January 1st, 1990 to December 31th, 1993. In the same way, the value corresponding to 1995 assumes knowledge from 1991 to 1994, and the rest of the values are obtained moving the window. Figures 21 - 25 do the same considering windows of two years, one year and six months respectively.

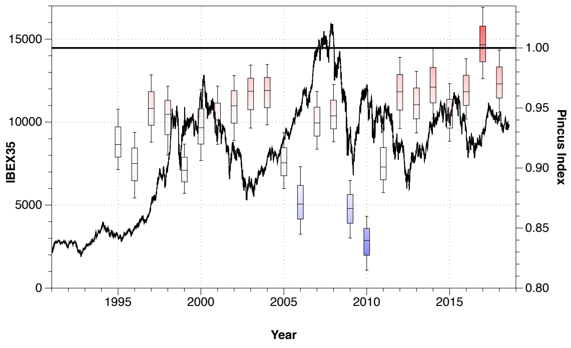

(a) Pincus Index of SampEn for the IBEX 35 considering information from the previous four years and moving every year.

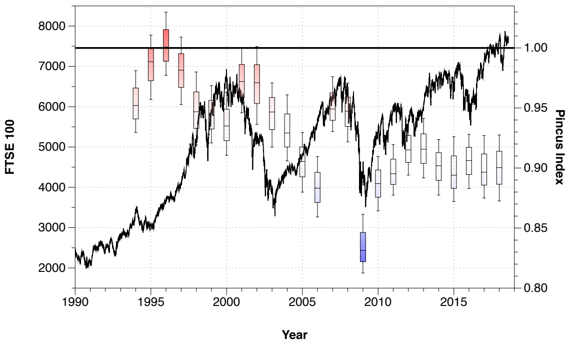

(b) Pincus Index of SampEn for the FTSE 100 considering information from the previous four years and moving every year.

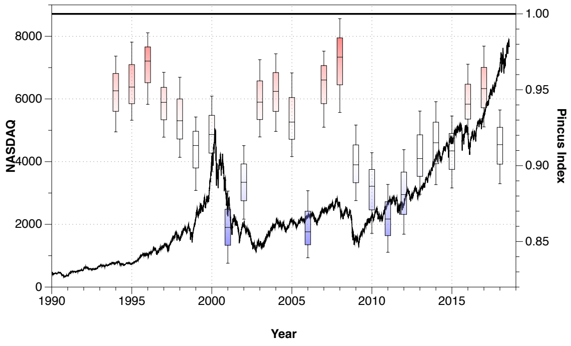

(c) Pincus Index of SampEn for the NASDAQ considering information from the previous four years and moving every year.

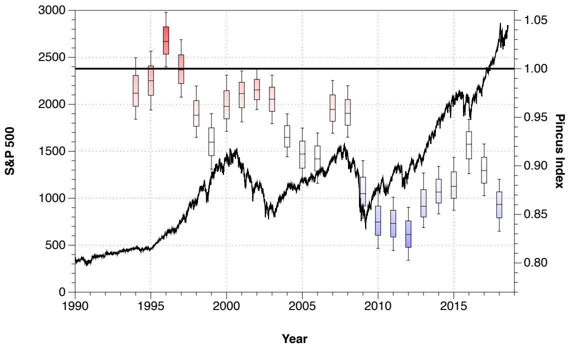

(d) Pincus Index of SampEn for the S&P 500 considering information from the previous four years and moving every year.

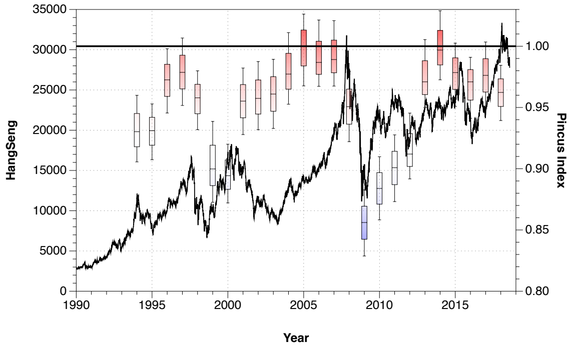

(e) Pincus Index of SampEn for the Hang Seng considering information from the previous four years and moving every year.

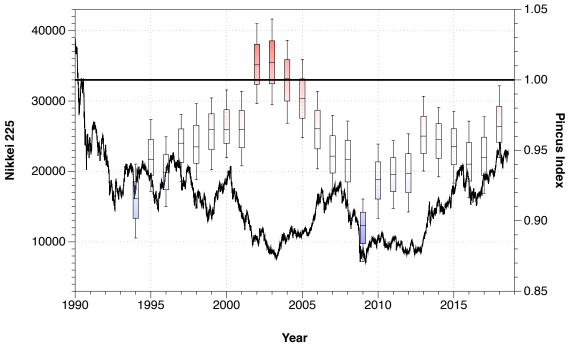

(f) Pincus Index of SampEn for the Nikkei 225 considering information from the previous four years and moving every year.

Figure 19: Pincus Index of SampEn for the different stock markets considering information from the previous four years and moving every year.

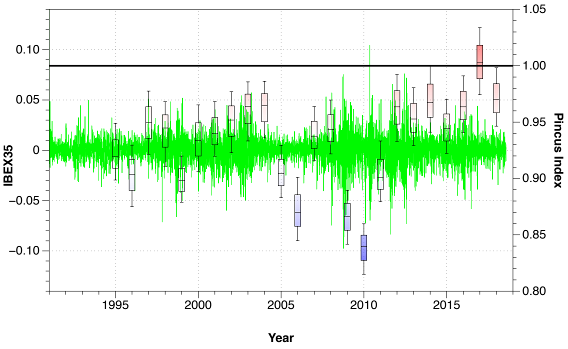

(a) Pincus Index of SampEn for the IBEX 35 considering information from the previous four years and moving every year.

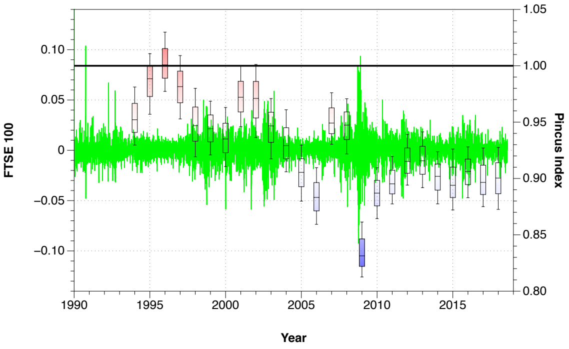

(b) Pincus Index of SampEn for the FTSE 100 considering information from the previous four years and moving every year.

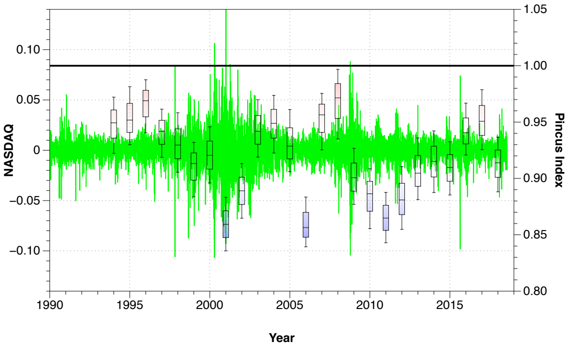

(c) Pincus Index of SampEn for the NASDAQ considering information from the previous four years and moving every year.

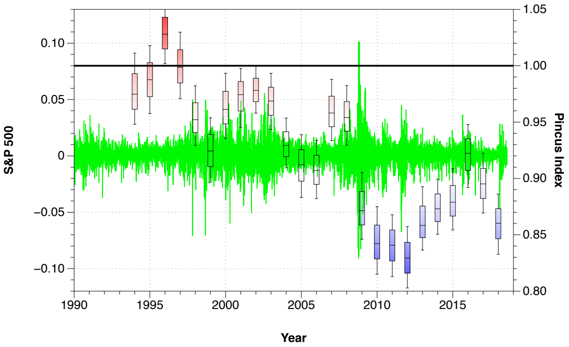

(d) Pincus Index of SampEn for the S&P 500 considering information from the previous four years and moving every year.

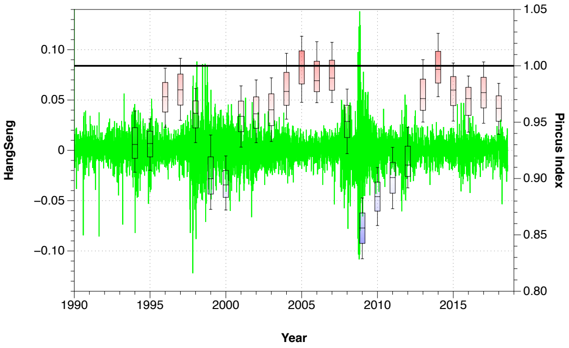

(e) Pincus Index of SampEn for the Hang Seng considering information from the previous four years and moving every year.

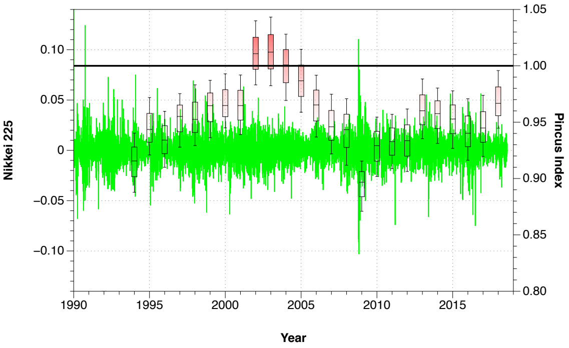

(f) Pincus Index of SampEn for the Nikkei 225 considering information from the previous four years and moving every year.

Figure 20: Pincus Index of SampEn for the different stock markets considering information from the previous four years and moving every year represented over their log-ratio series.

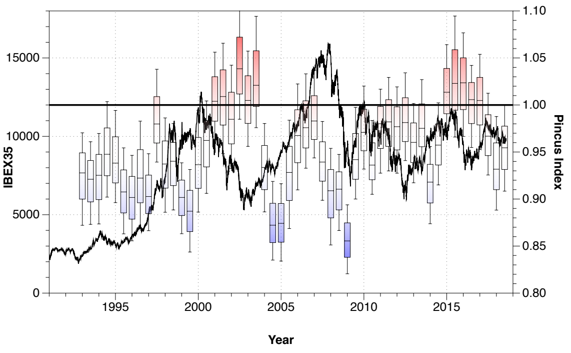

(a) Pincus Index of SampEn for the IBEX 35 considering information from the previous two years and moving every six months.

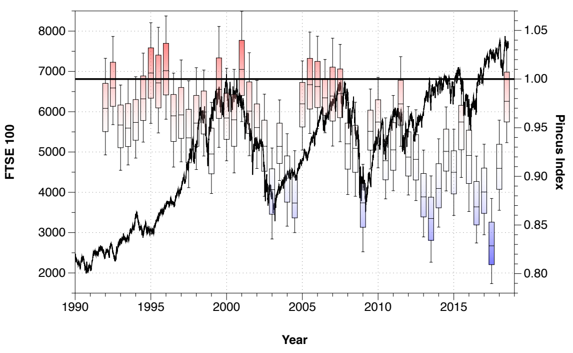

(b) Pincus Index of SampEn for the FTSE 100 considering information from the previous two years and moving every six months.

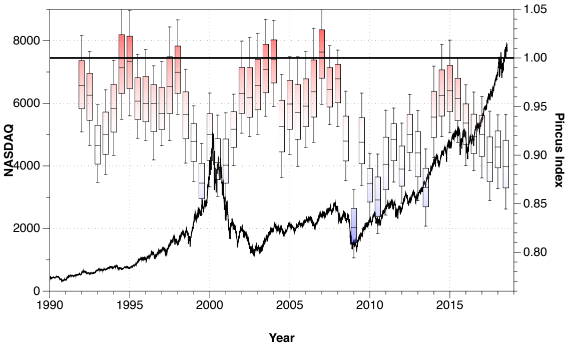

(c) Pincus Index of SampEn for the NASDAQ considering information from the previous two years and moving every six months.

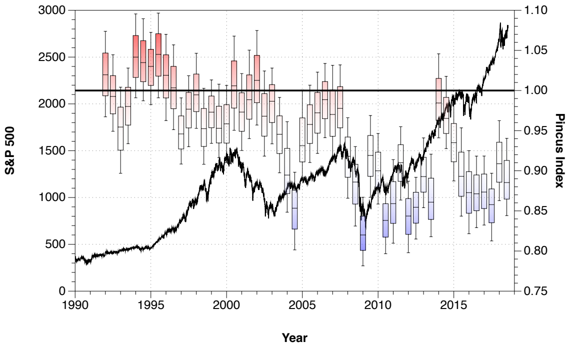

(d) Pincus Index of SampEn for the S&P 500 considering information from the previous two years and moving every six months.

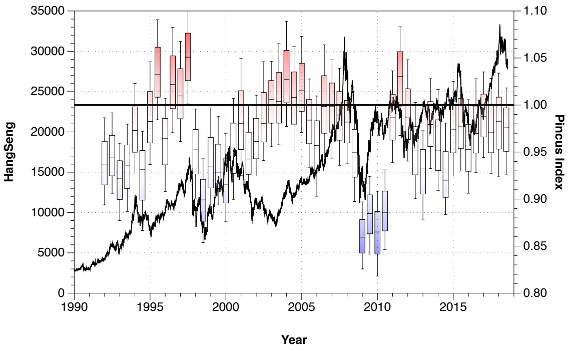

(e) Pincus Index of SampEn for the Hang Seng considering information from the previous two years and moving every six months.

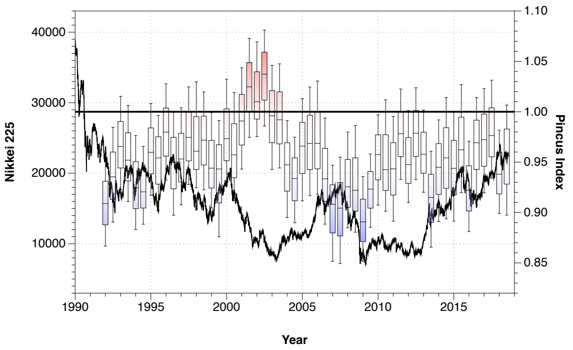

(f) Pincus Index of SampEn for the Nikkei 225 considering information from the previous two years and moving every six months.

Figure 21: Pincus Index of SampEn for the different stock markets considering information from the previous two years and moving every six months.

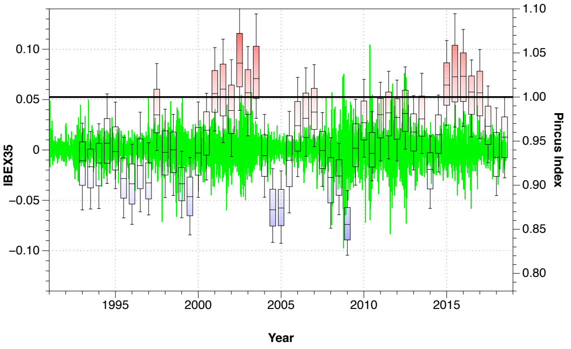

(a) Pincus Index of SampEn for the IBEX 35 considering information from the previous two years and moving every six months.

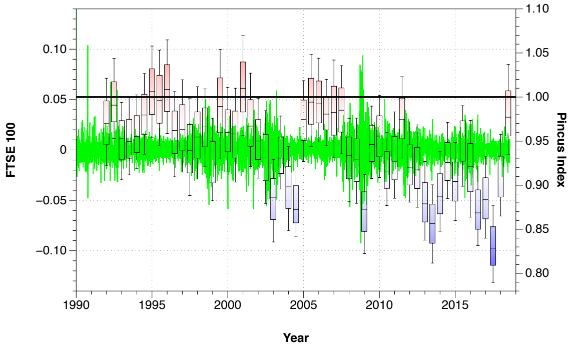

(b) Pincus Index of SampEn for the FTSE 100 considering information from the previous two years and moving every six months.

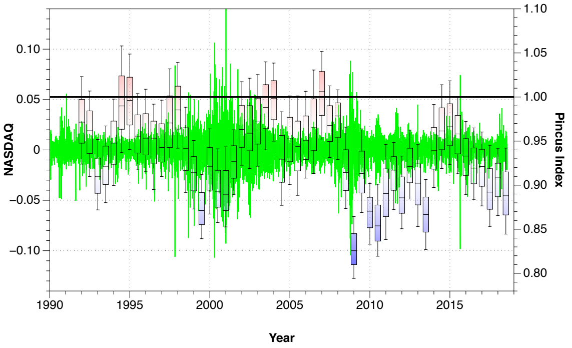

(c) Pincus Index of SampEn for the NASDAQ considering information from the previous two years and moving every six months.

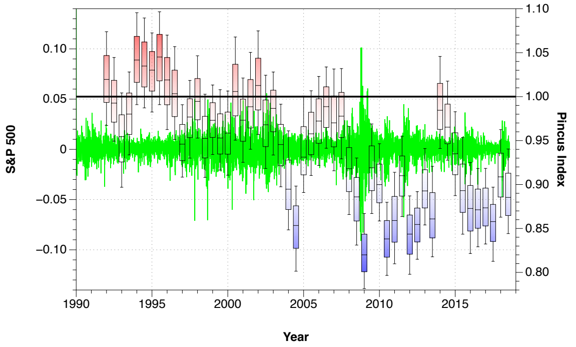

(d) Pincus Index of SampEn for the S&P 500 considering information from the previous two years and moving every six months.

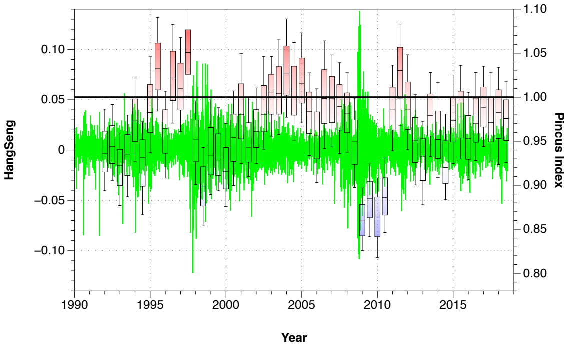

(e) Pincus Index of SampEn for the Hang Seng considering information from the previous two years and moving every six months.

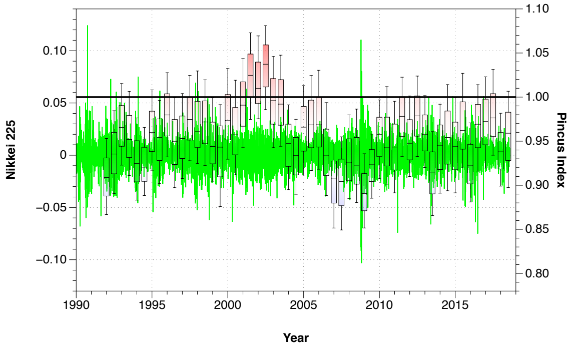

(f) Pincus Index of SampEn for the Nikkei 225 considering information from the previous two years and moving every six months.

Figure 22: Pincus Index of SampEn for the different stock markets considering information from the previous two years and moving every six months represented over their log-ratio series.

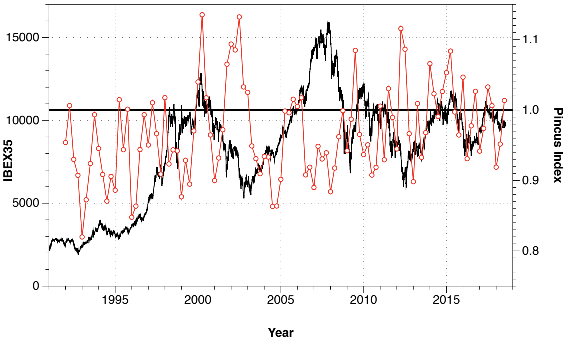

(a) Pincus Index of SampEn for the IBEX 35 considering information from the previous year and moving every three months.

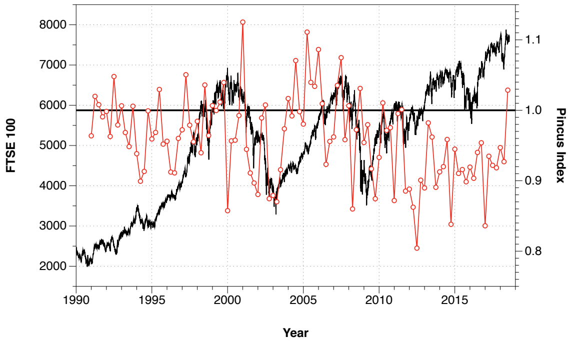

(b) Pincus Index of SampEn for the FTSE 100 considering information from the previous year and moving every three months.

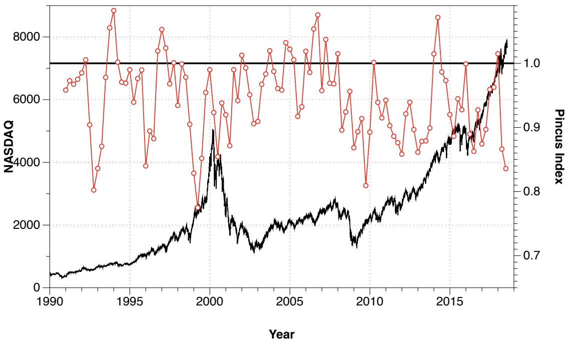

(c) Pincus Index of SampEn for the NASDAQ considering information from the previous year and moving every three months.

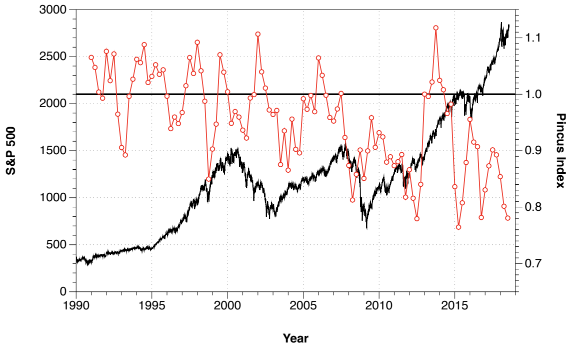

(d) Pincus Index of SampEn for the S&P 500 considering information from the previous year and moving every three months.

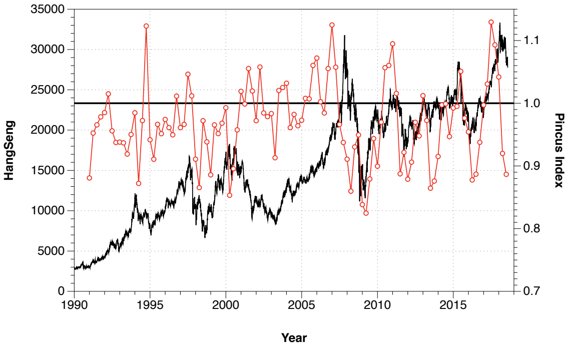

(e) Pincus Index of SampEn for the Hang Seng considering information from the previous year and moving every three months.

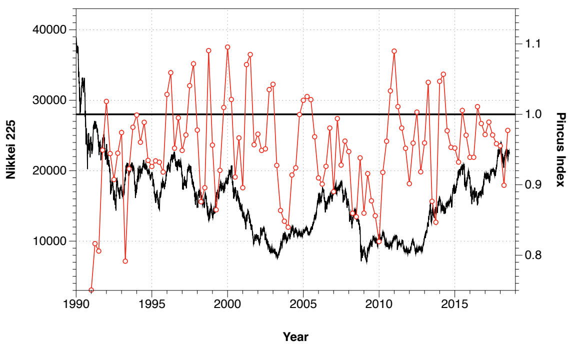

(f) Pincus Index of SampEn for the Nikkei 225 considering information from the previous year and moving every three months.

Figure 23: Pincus Index of SampEn for the different stock markets considering information from the previous year and moving every three months.

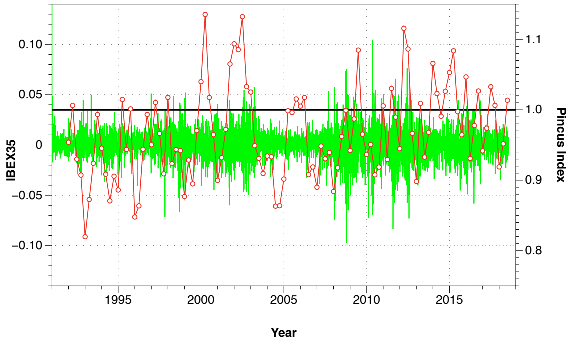

(a) Pincus Index of SampEn for the IBEX 35 considering information from the previous year and moving every three months.

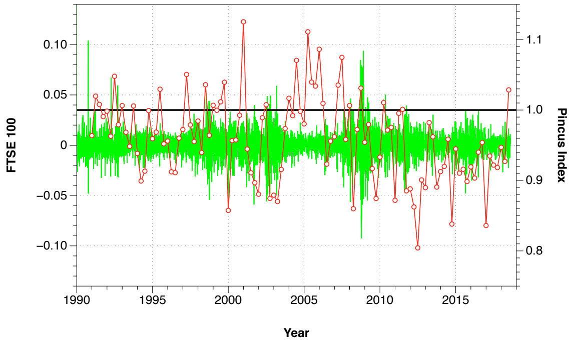

(b) Pincus Index of SampEn for the FTSE 100 considering information from the previous year and moving every three months.

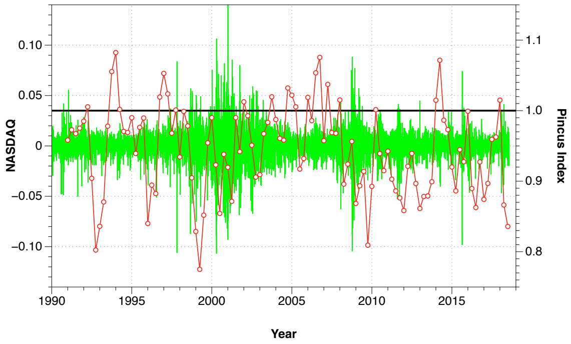

(c) Pincus Index of SampEn for the NASDAQ considering information from the previous year and moving every three months.

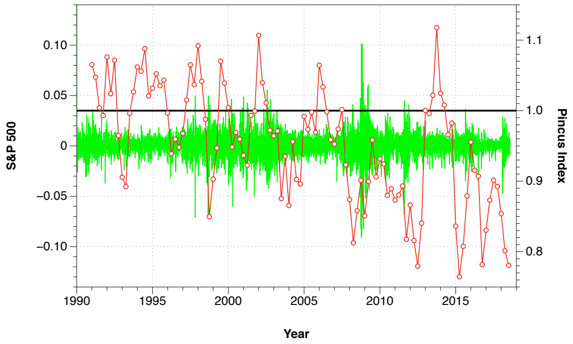

(d) Pincus Index of SampEn for the S&P 500 considering information from the previous year and moving every three months.

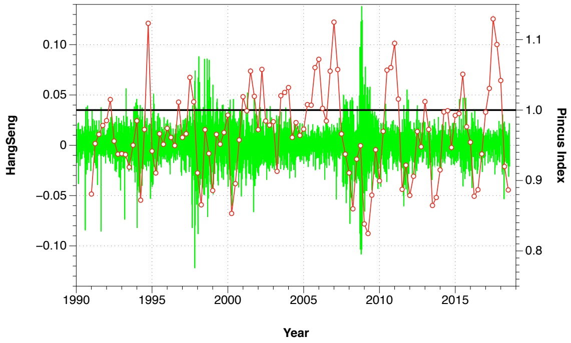

(e) Pincus Index of SampEn for the Hang Seng considering information from the previous year and moving every three months.

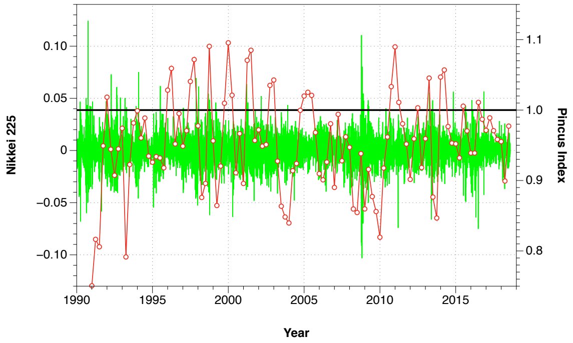

(f) Pincus Index of SampEn for the Nikkei 225 considering information from the previous year and moving every three months.

Figure 24: Pincus Index of SampEn for the different stock markets considering information from the previous year and moving every three months represented over their log-ratio series.

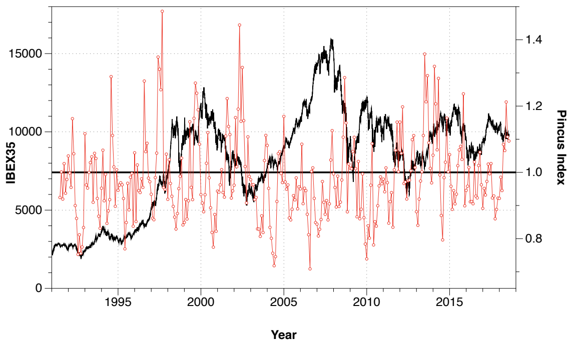

(a) Pincus Index of SampEn for the IBEX 35 considering information from the previous six months and moving every month.

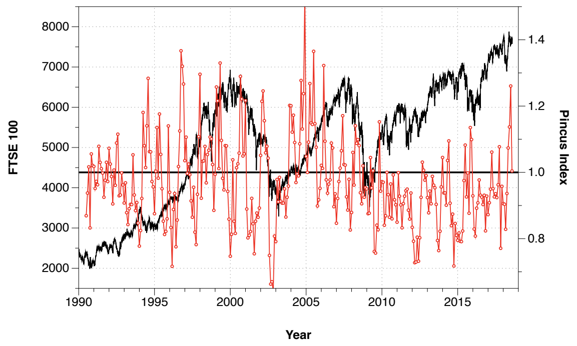

(b) Pincus Index of SampEn for the FTSE 100 considering information from the previous six months and moving every month.

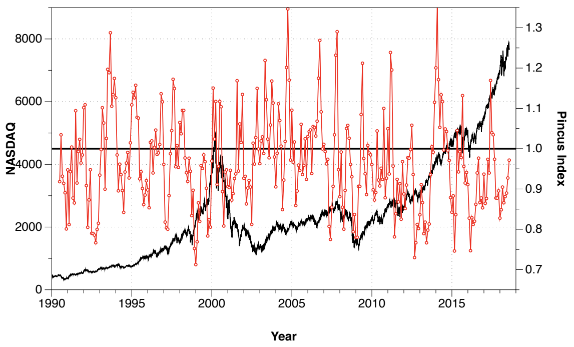

(c) Pincus Index of SampEn for the NASDAQ considering information from the previous six months and moving every month.

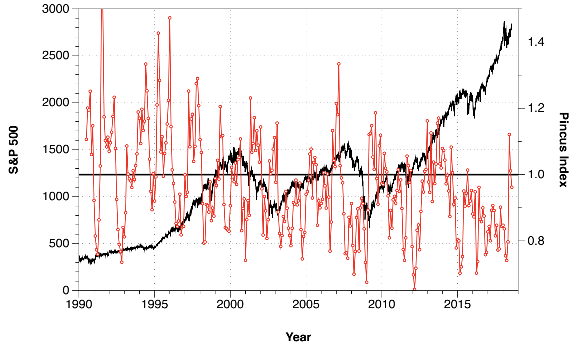

(d) Pincus Index of SampEn for the S&P 500 considering information from the previous six months and moving every month.

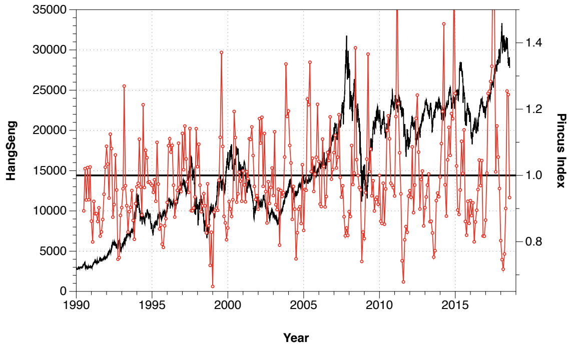

(e) Pincus Index of SampEn for the Hang Seng considering information from the previous six months and moving every month.

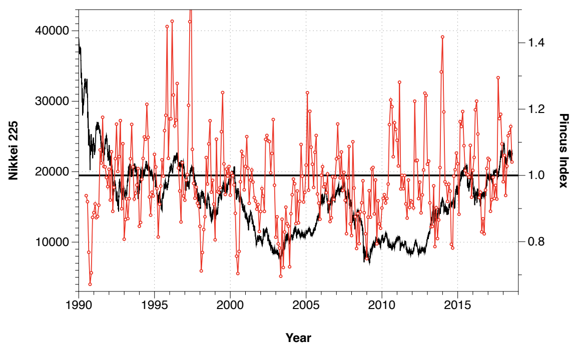

(f) Pincus Index of SampEn for the Nikkei 225 considering information from the previous six months and moving every month.

Figure 25: Pincus Index of SampEn for the different stock markets considering information from the previous six months and moving every month.

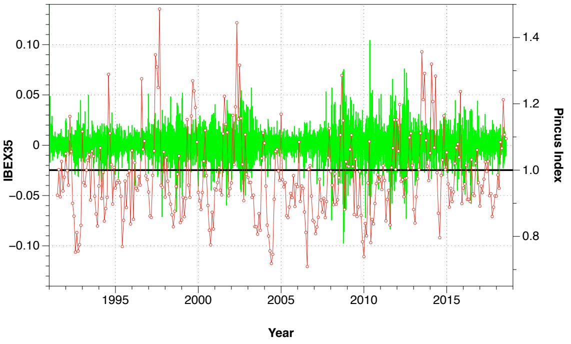

(a) Pincus Index of SampEn for the IBEX 35 considering information from the previous six months and moving every month.

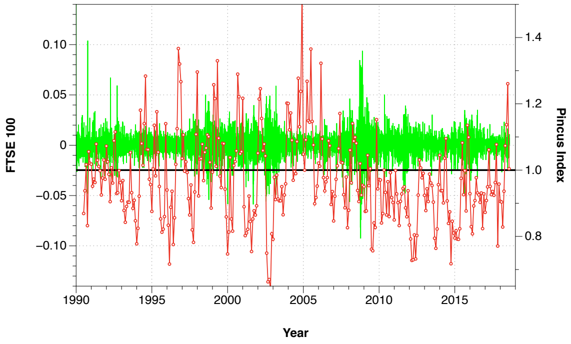

(b) Pincus Index of SampEn for the FTSE 100 considering information from the previous six months and moving every month.

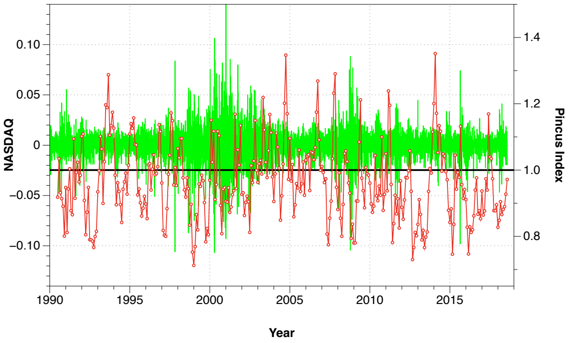

(c) Pincus Index of SampEn for the NASDAQ considering information from the previous six months and moving every month.

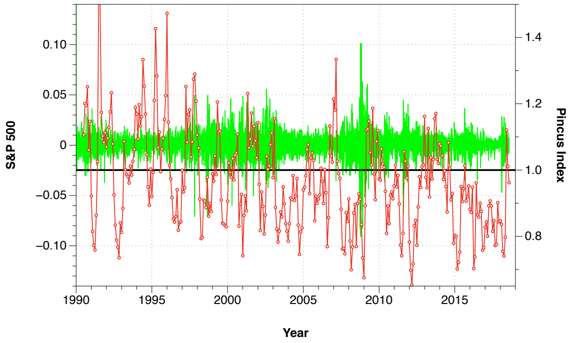

(d) Pincus Index of SampEn for the S&P 500 considering information from the previous six months and moving every month.

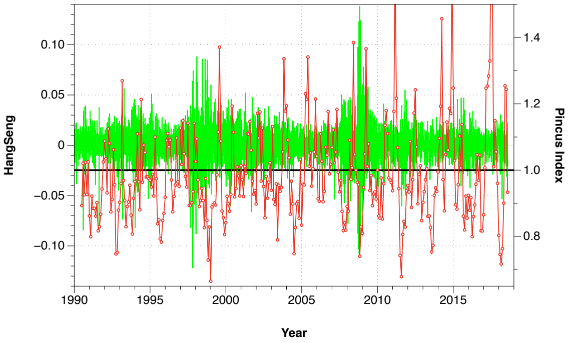

(e) Pincus Index of SampEn for the Hang Seng considering information from the previous six months and moving every month.

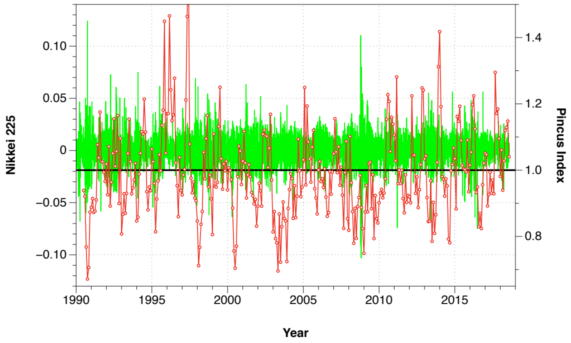

(f) Pincus Index of SampEn for the Nikkei 225 considering information from the previous six months and moving every month.

Figure 26: Pincus Index of SampEn for the different stock markets considering information from the previous six months and moving every month represented over their log-ratio series.
